# Genome sequencing and analysis of *Bacillus velezensis* VJH504 reveal biocontrol mechanism against cucumber *Fusarium* wilt

**DOI:** 10.3389/fmicb.2023.1279695

**Published:** 2023-10-12

**Authors:** Fan Yang, Huayan Jiang, Kai Ma, Xin Wang, Shen Liang, Yuxin Cai, Yancai Jing, Baoming Tian, Xuanjie Shi

**Affiliations:** ^1^Institute of Horticulture, Henan Academy of Agricultural Sciences, Graduate T&R Base of Zhengzhou University, Zhengzhou, Henan, China; ^2^School of Agricultural Sciences, Zhengzhou University, Zhengzhou, China

**Keywords:** *Fusarium oxysporum f*. sp. *cucumerinum*, *Bacillus velezensis*, biocontrol agent, growth promotion, genome sequencing and assembly, comparative genomic analysis, biocontrol mechanism

## Abstract

One major issue in reducing cucumber yield is the destructive disease Cucumber (*Cucumis sativus* L.) wilt disease caused by *Fusarium oxysporum* f. sp. *cucumerinum* (*Foc*). When using the isolate VJH504 isolated from cucumber rhizosphere soil and identified as *Bacillus velezensis*, the growth of *Foc* in the double culture experiment was effectively inhibited. Phenotypic, phylogenetic, and genomic analyses were conducted to enhance understanding of its biocontrol mechanism. According to the result of the phenotype analysis, *B. velezensis* VJH504 could inhibit cucumber *fusarium* wilt disease both *in vitro* and *in vivo*, and significantly promote cucumber seed germination and seedling growth. Additionally, the tests of growth-promoting and biocontrol characteristics revealed the secretion of proteases, amylases, β-1,3-glucanases, cellulases, as well as siderophores and indole-3-acetic acid by *B. velezensis* VJH504. Using the PacBio Sequel II system, we applied the complete genome sequencing for *B. velezensis* VJH504 and obtained a single circular chromosome with a size of 3.79 Mb. A phylogenetic tree was constructed based on the 16S rRNA gene sequences of *B. velezensis* VJH504 and 13 other Bacillus species, and Average Nucleotide Identity (ANI) analysis was performed using their whole-genome sequences, confirming isolateVJH504 as *B. velezensis*. Following this, based on the complete genome sequence od *B. velezensis* VJH504, specific functional analysis, Carbohydrate-Active Enzymes (CAZymes) analysis, and secondary metabolite analysis were carried out, predicting organism’s abilities for biofilm formation, production of antifungal CAZymes, and synthesis of antagonistic secondary metabolites against pathogens. Afterwards, a comparative genomic analysis was performed between *B. velezensis* VJH504 and three other *B. velezensis* strains, revealing subtle differences in their genomic sequences and suggesting the potential for the discovery of novel antimicrobial substances in *B. velezensis* VJH504. In conclusion, the mechanism of *B. velezensis* VJH504 in controlling cucumber *fusarium* wilt was predicted to appear that *B. velezensis* VJH504is a promising biocontrol agent, showcasing excellent application potential in agricultural production.

## 1. Introduction

Cucumber (*Cucumis sativus* L.) is one of the most important vegetable crops all over the world (Bartholomew et al., 2022). *Fusarium* wilt is a worldwide stubborn soil-borne disease, known as the “cancer” in plants. It is particularly difficult to control because of its persistence and universality, which seriously threatens the yield and quality of cucumber worldwide (Zhou et al., 2017). In addition to the reduction of cash crops, toxic secondary metabolites such as dehydrofumaric acid will be produced and left in crops, which will cause harm to humans and animals (Srinivas et al., 2019). Cucumber *fusarium* wilt is a fungal disease caused by *Fusarium oxysporum* f. sp. *cucumerinum* (*Foc*). Due to the ability of *Foc* to produce chlamydospores and conidia, which have strong resistance to adverse conditions, it is nearly impossible for *Foc* to be completely eliminated once it colonizes in soil (Zhao et al., 2017). Excessive use of chemical fungicides can lead to drug resistance of pathogen, causing damage to the ecological environment, and then posing a threat to human health. Therefore, safe and effective methods to control cucumber *fusarium* wilt are urgently needed.

Soil microorganisms are considered to be a key determinant of crop growth and health, and their typical role is to inhibit disease (Wen et al., 2023). In recent years, biological control has become a research hotspot in the field of sustainable agriculture because of its clear targeting, safety and pollution-free. *Bacillus* is a widely distributed biocontrol bacterium in nature. It is widely used in scientific research and production because of its large population, strong reproductive ability, stable physical and chemical properties and wide antibacterial spectrum. For example, *B. subtilis* (Xu et al., 2022), *B. velezensis* (Luo et al., 2019), *Paenibacillus polymyxa* (Li et al., 2014) and *B. amyloliquefaciens* (Wu Y. et al., 2015) could significantly inhibit or even destroy the mycelial growth and reduce the incidence of *F. oxysporum*. In addition, it could also induce the host to develop systemic resistance to *F. oxysporum*. *B. subtitls* YB-04 isolated from wheat straw, which is a promising biocontrol agent against cucumber *fusarium* wilt (Xu et al., 2022). It has strong antagonistic activity against *Foc* and obvious growth-promoting effect on cucumber seedlings. Medeiros and Bettiol (2021) found that *B. velezensis* AP-3 could promote the growth of tomato plants under salt stress, and protect tomato from *fusarium* wilt. Song et al. (2011) found the *P. polymyxa* isolated from the rhizosphere of rice has a wide antagonistic spectrum. The control effect of *P. polymyxa* on watermelon seedling *fusarium* wilt reached 52.8%, while the control effect of carbendazim commonly used in field watermelon *fusarium* wilt control was only 22.6%. At the same time, the seed germination rate was increased through *P. polymyxa*. Yuan et al. (2012) studied an antifungal activity of *B. amyloliquefaciens* NJN-6 volatile compounds against banana *fusarium* wilt, and found that 11 volatile compounds could completely inhibit the growth of banana *fusarium* wilt.

As an excellent biocontrol bacterium, *B. velezensis* has been successfully applied to the prevention and treatment of various plant diseases, which has the characteristics of wide antibacterial spectrum, strong stress resistance and diverse biocontrol mechanisms. And it is harmless to humans and animals, does not pollute the environment. In recent years, there have been more and more reports on *B. velezensis* at home and abroad, mainly focusing on antagonistic pathogens (Liu et al., 2017; Zhou et al., 2023), promoting plant growth (Kong et al., 2018; Fan et al., 2019; Afzal et al., 2023), inducing plant systemic resistance (Harun-Or-Rashid et al., 2017; Jiang et al., 2019; Rabbee et al., 2019) and other defense responses. *B. velezensis* has obvious antagonistic activity against *F. oxysporum*, *F. graminearum*, *Rhizoctonia cerealis*, *Botryosphaeria dothidea*, *Verticillium dahliae* and other plant pathogens (Zong et al., 2018; Zhang Q. et al., 2020; Li et al., 2021). The strong antibacterial spectrum of *B. velezensis* has been benefited from various kinds of antibacterial mechanisms mediated by many of secondary metabolites, such as competition, lysis, antagonism (Zhang et al., 2020a). *B. velezensis* can inhibit the growth of pathogens by synthesizing lipopeptides, polyketides, bacteriocins and antifungal proteins through polyketide synthase synthesis pathway, ribosomal pathway and non-ribosomal pathway (Zhang et al., 2020a; Xie et al., 2021). Strain YB15 secretes antibacterial substances such as β-glucanase, which plays an antagonistic role against pathogenic fungi (Xu et al., 2014). Strain SH-1471 has *srfA*, *fenB*, *ituA*, *ituD* and *bymA* genes, which can inhibit the growth of fungi and bacteria and induce the formation of biofilm by synthesizing a variety of lipopeptide antibiotics. In addition, the siderophore synthesis ability of *SH-1471* is also an important way to induce plant systemic resistance to resist pathogen infection. It can promote plant root system development and nutrient uptake by secreting siderophore to promote plant growth and induce systemic disease resistance. Recent studies have also shown that most of *B. velezensis* can secrete cyclic lipopeptides such as surfactin, iturin and fengycin, which act as elicitors to stimulate systemic induced resistance in plants (Lam et al., 2021; Stoll et al., 2021).

*Bacillus velezensis* is widely distributed and grows rapidly, and has the ability to produce a large amount of secondary metabolites such as antimicrobial proteins and lipopeptide antibiotics. Therefore, it is an excellent biocontrol resource (Zhang et al., 2020a). However, the morphology and physicochemical properties of strains from different sources are not the same. Some strains have single function, and lack of diversified functions for development and utilization. Complete genome sequence is a technology that randomly interrupts, sequences and splices the genome of organisms to obtain a complete genome sequence. Through sequence analysis and functional gene annotation (including NR, KEGG, GO, and CARD), the mechanism of strain phenotype can be deeply understood at the gene level (Goodwin et al., 2016; Galperin et al., 2019). *FZB42* is one of the model strains of *B. velezensis*. Its complete genome sequencing and functional prediction results show that about 10% of the gene resources are used to produce various secondary metabolites, which have a wide range of pathways and diverse types. These secondary metabolites have varying degrees of antibacterial and plant growth-promoting abilities. Experiments have also proved that this strain has powerful inhibitory effects on dozens of plant pathogenic bacteria and pathogenic fungi (Li et al., 2016). Zhou et al. (2023) isolated *B. velezensis* GUAL210 from the rhizosphere of healthy pepper plants grown in anthracnose-diseased fields in Guizhou, China, and sequenced its whole genome to predict that its biocontrol mechanism may be secondary metabolites with antifungal activity. It has been reported that the complete genome of *B. velezensis* C4341 isolated from saline-alkali soil in Xinjiang, China, that is composed of 4,019 open reading frames (ORFs), of which 5.9 and 1.6% are related to antagonistic secondary metabolites and antibiotic resistance, respectively (Zhu et al., 2019). In recent years, *B. velezensis* has received extensive attention from researchers in the field of agricultural pest and disease control. Current researches on *B. velezensis* mainly focus on isolation and identification, biocontrol effects, and gene functions. There are few reports on the screening of strains suitable for specific environment, biocontrol mechanism and colonization ability of *B. velezensis*. In this study, the highly active *B. velezensis* VJH504, isolated from cucumber rhizosphere soil, was selected as the research object. Using activity screening models such as the agar diffusion method and substrate degradation assay, the strain’s biocontrol activity against pathogenic microorganisms, as well as its activities in producing proteases, cellulases, pectinases, and iron carriers, were explored. Through the analysis of the gene sequence information of *B. velezensis* VJH504, the functional mechanisms of its biological control and plant growth promotion were further elucidated at the genomic level, providing a theoretical basis and guidance for the future development and application of this strain.

## 2. Materials and methods

### 2.1. Soil sample collection and separation and purification of bacteria

From the cucumber planting area in Beiwang Village, Luolong District, Luoyang City, Henan Province, which is a high-incidence area of cucumber wilt disease, soil samples were taken from the rhizosphere of healthy cucumber plants. One gram of soil sample was weighed into a 10 mL centrifuge tube and 9 mL of sterile water was added to suspend the soil sample thoroughly. The suspension was then diluted to a concentration of 10^−5^. 100 μL of the diluted suspension was taken and evenly spread on an LB agar plate. The plate was inverted and incubated at 30°C for 2 days. After incubation, bacterial colonies with different morphologies were selected and streaked for purification three times. The purified strains were stored in a refrigerator at 4°C for future use.

#### 2.1.1. Screening of soil antagonistic bacteria

The plate confrontation method was used to screen for antagonistic bacteria, using *Foc* FJH36 as the indicator strain. *Foc* FJH36 was isolated and preserved from the Institute of Horticulture, Henan Academy of Agricultural Sciences.

#### 2.1.2. Primary screening

Using a 5 mm diameter punch, a plug containing the pathogenic fungus (*Foc* FJH36) was taken from the edge of the *Foc* FJH36 agar plate and placed at the center of a Potato Dextrose Aga (PDA) plate. Bacteria were inoculated 25 mm away from the plug in a straight line. A control plate was prepared by inoculating *Foc* FJH36 without the bacteria. This process was repeated three times. The plates were incubated at 28°C for 7 days, and the diameter of the pathogenic fungus was measured to calculate the antimicrobial activity (Xu et al., 2020).

#### 2.1.3. Secondary screening

The strains with good antagonistic effects from the initial screening were subjected to a secondary screening using the hyphal growth rate method. Each selected bacterial strain was inoculated into 100 mL of Luria-Bertain (LB) medium. After incubating at 37°C and 160 rpm for 36 h, a biocontrol bacterial solution with OD_600_ = 2.0 was obtained. The solution was centrifuged at 4°C and 10,000 rpm for 10 min, and the supernatant was collected. The supernatant was then filtered three times through 0.22 μm sterile microporous filter to obtain a sterile fermentation broth of the strain. The sterile fermentation broth was added to cooled PDA medium at a volume ratio of 1:1, when the temperature was around 45°C. After pouring the mixture into petri dishes, 50 μL of *Foc* FJH36 spore suspension was added to each plate for coating. The control group consisted of only the *Foc* FJH36 spore suspension without the fermentation broth. After cultured at 28°C for 24 h, the spore germination was observed by microscope. After 48 h, the changes of mycelium were observed by microscope.

### 2.2. Pot experiment to test the biocontrol effect of *Bacillus velezensis* VJH504 on cucumber *fusarium* wilt and the effect of promoting germination and growth

#### 2.2.1. Preparation of fungal and bacterial suspensions

The *Foc* FJH36 was cultured on PDA plates at 28°C for 5 days. Then, using a 5 mm punch, 10 plugs containing the pathogenic fungus were transferred to 100 mL of Potato Dextrose Broth (PDB). The culture was incubated on a shaker at 28°C and 180 rpm for 3 days. Afterward, the *Foc FJH36* culture was filtered through four layers of sterile gauze, and sterile water was added to dilute the concentration of pathogenic fungal spores to 1 × 10^8^ CFU/mL.

The strain *B. velezensis* VJH504 was cultured in LB medium at 37°C and 180 rpm for 24 h. After the incubation, the culture was centrifuged at 4,000 xg for 5 min to collect the bacterial cells. The cells were then suspended in LB medium, and further diluted to a concentration of 1 × 10^8^ CFU/mL.

#### 2.2.2. Effect of *Bacillus velezensis* VJH504 on promoting cucumber seed germination

The healthy, plump, and uniformly sized cucumber seeds of the variety Bojie 616 were selected and surface sterilized with 75% ethanol (v/v) for 30 s, followed rinsing with sterile water for 5 times. The sterilized seeds were pre-soak in warm water at 55°C for 10 min. After removing excess water from the seed surface using filter paper, the seeds were immersed in a bacterial suspension of *B. velezensis* VJH504 for 4 h and rinsed with sterile water for three times. For the control treatment, the seeds were soaked in distilled water for the same duration. The seeds were placed in petri dishes lined with two layers of qualitative filter paper. Each dish contained 40 seeds, and each treatment repeated the process for three times. The filter paper was kept moisture with water and the dishes were placed in a growth chamber at a temperature of 30°C and humidity between 60 to 70%. After 24 h, the germination of the seeds in each treatment was examined and the germination rate for each treatment was calculated.

#### 2.2.3. Effect of *Bacillus velezensis* VJH504 on the growth of cucumber seedlings

Bojie 616 cucumber seeds with strong, full and uniform size were selected. The seeds were disinfected with 75% ethanol (v/v) for 30 s and then rinsed with sterile water for 5 times. The seeds were soaked in warm water (55°C) for 10 min. Then soaked in distilled water for 4 h. Wet a sterile cloth with sterile water and rinse the soaked cucumber seeds several times. The washed seeds were neatly arranged on the cloth and placed in a constant temperature incubator at 28°C for germination. Once the seed has germinated and shows a white tip, it is sown in a sterile substrate-filled seedling tray. Six days after sowing, the seedlings with two true leaves were transferred to a pot with a diameter of 10 cm and a height of 10 cm. The pot was filled with 400 g sterile substrate. Five pots of plants were placed in a greenhouse at a temperature of 14 ~ 25°C for 16 h of light and 8 h of darkness, with a light cycle of 4 d. The experiment was conducted using a completely randomized design with five treatments and five replications per treatment. The treatments were as follows: (1) Root irrigation with 20 mL of *B. velezensis VJH504* suspension every 2 days for a total of three irrigations; (2) After three root irrigations with 20 mL of *B. velezensis* VJH504 suspension, root irrigation with 20 mL of *Foc FJH36* spore suspension; (3) Root irrigation with 20 mL of 0.1% hymexazol once, followed by root irrigation with 20 mL of *Foc FJH36* spore suspension after 24 h; (4) Root irrigation with 20 mL of sterile distilled water every 2 days for a total of three irrigations; (5) After three root irrigations with 20 mL of sterile distilled water, root irrigation with *Foc FJH36* spore suspension. Measurements were taken at 20 and 40 days after inoculation of the pathogen. The measured parameters included plant height, stem thickness, leaf area, chlorophyll content, aboveground fresh weight, belowground fresh weight, aboveground dry weight, belowground dry weight. The vigor index was calculated as (stem thickness/plant height + root dry weight/aboveground dry weight) × total plant dry weight. The severity and incidence of cucumber wilt disease were recorded at 40 days. Cucumber wilt disease was classified into 5 levels: 0 = no symptoms on leaves; 1 = wilting of leaves less than 1/4 of cucumber seedlings; 2 = wilting of 1/4 to 1/2 of cucumber seedling leaves; 3 = wilting of more than 1/2 of cucumber seedling leaves; 4 = complete wilting and death of the whole cucumber plant. Disease index (DI) was calculated using the formula DI = [(0 × N0) + (1 × N1) + (2 × N2) + (3 × N3) + (4 × N4)]/(T × 4) × 100, where N represents the number of cucumber seedlings with each disease rating, and T is the total number of cucumber seedlings. Disease incidence was calculated as [(N1 + N2 + N3 + N4)/T] × 100%. Control efficacy was determined by the formula (Control DI - Treatment DI)/Control DI × 100%. Leaf chlorophyll content was measured using an SPAD-502 Plus chlorophyll meter. Root length and branch height were measured using a ruler. Root and stem fresh weights were recorded using an analytical balance (ME203E, Mettler Toledo, Shanghai, China). Stem thickness at a distance of 2 cm from the crown was measured using a caliper (MNT-200, Shanghai Mettler Instrument Co., Ltd., Shanghai, China).

### 2.3. Detection of plant growth-promoting bacteria and biological control agents traits

*Bacillus velezensis* VJH504 was inoculated in skim milk agar (0.1 g Ca Cl_2_, 5.0 g Na Cl, 10.0 g peptone, 18.0 g agar, pH 7.2) with a single colony, and cultured at 30°C for 48 h (Kazanas, 1968). The clear area around the colony proved that the strain had protease activity. Amylase activity was detected with single colonies grown at 30°C for 48 h on starch agar (10.0 g soluble starch, 10.0 g pancreatic digest of casein, 5.0 g glucose, 5.0 g NaCl, 5.0 g beef extract, and 18.0 g agar per liter, pH 7.2). Add Lugol’s iodine solution (1% iodine in 2% potassium iodide w/v) to the starch agar plates, ensuring an even spread across the plate. The colorless halos around the colony proved that the strain had amylase activity (Al-Naamani et al., 2015). *B. velezensis* VJH504 was inoculated into carboxymethyl cellulose (CMC) agar (5.0 g CMC-Na, 0.1 g MgSO4·7H2O, 0.25 g K2HPO4, 18.0 g agar / L, pH 5.5) with single colony, and cultured at 30°C for 48 h. Flood the plate with 1% (w/v) Congo Red solution, and then rinse with sterile distilled water. The transparent area around the colony proved that the strain had cellulase activity. Inoculate *B. velezensis* VJH504 as single colonies in Chrome Azurol S (CAS) blue agar (10 mL 20% sucrose solution, 30 mL 10% acid-hydrolyzed casein, 1 mL 1 mmol/L CaCl_2_, 5 mL 0.1 mol/L phosphate buffer saline (pH 6.8), 50 mL CAS staining solution, and 18 g agar / L, pH 7.2), and culture at 30°C for 48 h. A color change from blue to orange around the colonies indicates the production of siderophores. *B. velezensis* VJH504 was inoculated into L-tryptophan nutrient broth (3 g beef extract, 10 g peptone, 5 g Na Cl, 0.5 g L-tryptophan / L, pH 7.2) with a single colony, and cultured at 30°C for 48 h. After centrifugation at 14,000 xg for 10 min, mix 1 mL of the supernatant with 2 mL of Salkowski reagent, and then allow it to stand at room temperature in the dark for 30 min. A color change from yellow to orange indicates the production of indole-3-acetic acid (IAA) (Glickmann and Dessaux, 1995). *B. velezensis* VJH504 was inoculated in β-glucan agar (0.05 g glucose, 0.5 g yeast extract, 1 g peptone, 0.5 g NaCl, 0.01 g Congo red, 18.0 g agar / L, pH 7.0) and cultured at 30°C for 48 h. The transparent area around the colony proved that the strain had β-glucanase activity (Teather and Wood, 1982).

### 2.4. Determination of defense enzyme activities in the cucumber leaves

After 20 dpi of strain inoculation, the leaves were harvested and stored at −80°C. 0.5 g of leaves were ground in liquid nitrogen and 1 mL of extraction buffer was added. After centrifugation at 8000 xg for 10 min, the supernatant was collected for enzyme determination. Enzyme activities were measured using assay kits for PPO, SOD, CAT, PAL, and LOX following the manufacturer’s protocols (Solarbio, Beijing, China). Absorbance was measured using a microplate reader (Tecan Spark, Tecan, Switzerland).

### 2.5. The DNA extraction, genome sequencing, and assembly of *Bacillus velezensis* VJH504

The single colony of *B. velezensis* VJH504 was inoculated in LB medium and cultured at 28°C, 150 rpm for 18 h. The genomic DNA of *B. velezensis* VJH504 is extracted using the Mini BEST Bacteria Genomic DNA Extraction Kit Ver.3.0 according to the manufacturer’s instructions. A 10 kb insert fragment Pac Bio library was constructed and the whole genome was sequenced using the Pac Bio Sequel II system. The sequencing reads are assembled *de novo* using HGAP4 and Canu software. The genome coverage depth is analyzed using alignment tools. The assembled complete genome sequence of *B. velezensis* VJH504 is deposited in NCBI GenBank under accession number CP131928. A circular genome map of *B. velezensis* VJH504 is constructed using Circos (Krzywinski et al., 2009).

### 2.6. Genome annotation of *Bacillus velezensis* VJH504

The genome of *B. velezensis* VJH504 was annotated using Glimmer (v3.02) (Delcher et al., 2007). tRNA and rRNA genes were identified using tRNAscan-SE (v2.0) (Lowe and Eddy, 1997) and RNAmmer (v1.2) (Lagesen et al., 2007), respectively. BLASTx was performed against the NCBI non-redundant protein database (NR), Swiss-Prot, Clusters of Orthologous Groups (COG), Kyoto Encyclopedia of Genes and Genomes (KEGG), and Gene Ontology (GO) for functional annotation.

### 2.7. Analysis and identification of *Bacillus velezensis* VJH504

A phylogenetic tree based on the 16S rRNA gene sequences of *B. velezensis* VJH504 and *B. velezensis* FZB42, *B. velezensis* G341, *B. velezensis* BvL03, *B. subtilis* YB-04, *B. subtilis* H1, *B. amyloliquefaciens* 35 M, *B. amyloliquefaciens* Ba13, *B. licheniformis* ATCC 14580, *B. licheniformis* SRCM 120569, *B. pumilus* ZB201701, *B. pumilus* SF-4, *B. thuringiensis* B13, and *B. thuringiensis* JW-1 (GenBank IDs: CP000560.2, NZ_CP011686.1, NZ_CP041192.1, NZ_CP072525.1, CP026662.1 CP082278.1, CP073635.1, CP034569.1, CP035404.1, CP029464.1, CP047089.1, CP074714.1, NZ_CP045030.1) was constructed using the Neighbor Joining method in MEGA7.0 (Kumar et al., 2016). The average nucleotide identity (ANI) was calculated using the ANI calculator (Yoon et al., 2017).

### 2.8. Analysis of CAZymes and secondary metabolic genes of *Bacillus velezensis* VJH504

Using dbCAN2 (Zheng et al., 2023) and HMMER (v3.1b2) (Finn et al., 2011), the predicted protein sequences from the genome of *B. velezensis* VJH504 are aligned against the Carbohydrate-Active Enzymes (CAZy) database (Lombard et al., 2014) with an E-value threshold of 1e^−15^. The signal peptides of identified CAZymes are analyzed using SignalP (v4.1) (Petersen et al., 2011). The protein-coding genes in the genome of *B. velezensis* VJH504 are compared to the CAZy database and analyzed for secondary metabolites using antiSMASH version 7.0.0 (Blin et al., 2019).

### 2.9. Statistical analysis

Statistical analysis was performed using SPSS v21.0. One-way analysis of variance (ANOVA) was conducted to analyze the data. The mean values were compared using Duncan’s multiple range test with a significance level set at *p* ≤ 0.05.

## 3. Results

### 3.1. Isolation of *Bacillus velezensis* VJH504 strain and its biocontrol activity against *Fusarium* wilt *in vitro* and *in vivo*

**Figure 1:**
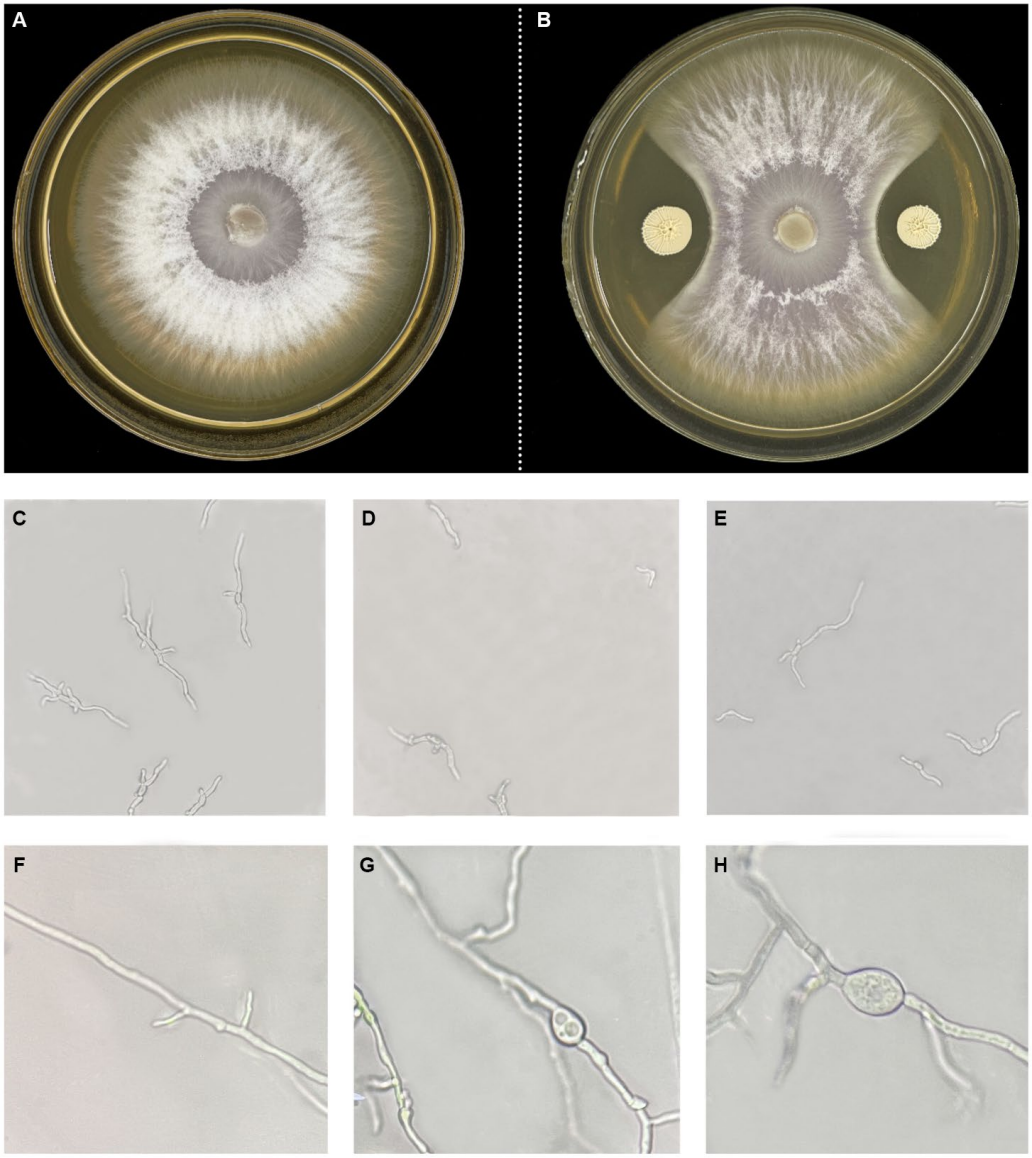
Colony and mycelial morphology of *Foc* FJH36 with or without *B. velezensis* VJH504. **(A,B)** Colony morphology of *Foc* FJH36 on PDA plate without and with *B. velezensis* VJH504, respectively; **(C)** Spores germination status of *Foc* FJH36 on PDA plate; **(D,E)** Microscopic photos of spore germination of *Foc* FJH36 treated with fermentation broth of *B. velezesis* VJH504; **(F)** Mycelial morphology of *Foc* FJH36 on PDA plate; **(G,H)** Microscopic photos of mycelial morphology of *Foc* FJH36 treated with fermentation broth of *B. velezensis* VJH504.

To perform the activity test against *Foc* FJH36 in the dual-culture assay, twelve strains were chosen from the numerous colonies with various morphologies, isolated and purified from healthy cucumber rhizosphere soil (Table 1). *B. velezensis* VJH504 was chosen for additional investigation basease it displayed the maximum antagonistic activity against *Foc* in culture media (Figures 1A,B), with an antagonistic zone width of 10.17 mm and an inhibition rate of 86.40%. The results of re-screening showed that the spore germination of fungi was slower than that of the control treatment after *Foc* was cultured in the medium containing *B. velezensis* VJH504 for 24 h (Figures 1C,D). After 48 h, the hyphae of *Foc* were observed to expand significantly (Figures 1E,F). The severity of *fusarium* wilt of cucumber seedlings treated with *B. velezensis* VJH504 was reduced at 20 dpi after inoculation with *Foc*. By observing the phenotypic indexes of cucumber seedlings treated with five treatments for 20 dpi and 40 dpi, we found that the indexes of *Foc* + 0.1% Hymexazol, *Foc* + VJH504 and VJH504 treatments were significantly higher than those of *Foc* treatment. In terms of plant height, stem diameter, leaf area, shoot fresh weight and seedling index, result of VJH504 and *Foc* + VJH504 treatments were significantly higher than those of *Foc* + 0.1% Hymexazol treatment (Table 2). At the same time, the 40 dpi disease incidence, disease index and control effect showed that VJH504 significantly reduced the wilt symptoms caused by *Foc*, and its level was slightly lower than that of the chemical fungicide hymexazol (Table 3).

**Table 1:** Antagonistic activity of 12 strains against *Foc* in dual culture.

Antagonistic bacteria	Antagonism band width (mm)	Antibacterial rate
NF9	1.97 ± 0.35^g^	16.40%^l^
B1	3.03 ± 0.55^f^	26.45%^k^
NF2	5.1 ± 0.17^e^	28.57%^j^
PJH16	8.03 ± 0.15^b^	44.24%^g^
D21	7.07 ± 0.40^c^	52.97%^e^
B124	6.00 ± 0.26^d^	55.66%^d^
A9	7.13 ± 0.15^c^	74.67%^b^
A52	6.17 ± 0.31^d^	72.33%^c^
VJH504	10.17 ± 0.30^a^	86.40%^a^
A5	6.07 ± 0.25^d^	32.69%^i^
A35	7.97 ± 0.32^b^	48.71%^f^
B67	6.00 ± 0.26^d^	35.33%^h^

Within the same column, different letters (a-l) indicate significant differences at *p* < 0.05 level.

**Table 2:** The impact of *B. velezensis* VJH504 on growth parameters of cucumber seedlings.

	Treatment	CK	*Foc*	*Foc* + 0.1% Hymexazol	*Foc* + VJH504	VJH504
20 dpi	Shoot height (cm)	5.85 ± 0.42^c^	4.33 ± 0.4^d^	6 ± 0.2^c^	7.28 ± 0.4^b^	7.87 ± 0.8^a^
	Stem thickness (mm)	3 ± 0.11^c^	2.08 ± 0.26^d^	3.27 ± 0.18^b^	3.48 ± 0.29^a^^b^	3.6 ± 0.09^a^
	Root length (cm)	13.75 ± 2.32^b^	5.16 ± 0.23^c^	17.13 ± 0.25^a^	18.01 ± 1.54^a^	17.75 ± 1.96^a^
	Leaf area 1 (cm^2^)	20.93 ± 0.6^d^	15.38 ± 0.82^e^	23.86 ± 0.67^c^	39.1 ± 2.24^b^	41.68 ± 3.41^a^
	Leaf area 2 (cm^2^)	2.36 ± 1.08^d^	0.48 ± 0.1^e^	4.22 ± 0.48^c^	7.92 ± 1.5^b^	11.43 ± 2.85^a^
	Chlorophyll content (SPAD)	45.77 ± 1.67^c^	32.83 ± 2.35^d^	50.45 ± 1.45^b^	59.3 ± 0.71^a^	60.84 ± 2.62^a^
	Shoot fresh weight (g)	1.31 ± 0.11^c^	0.54 ± 0.06^d^	1.9 ± 0.32^b^	2.14 ± 0.12^a^	2.24 ± 0.17^a^
	Root fresh weight (g)	0.74 ± 0.09^a^	0.24 ± 0.03^b^	0.86 ± 0.03^a^	0.88 ± 0.22^a^	0.83 ± 0.19^a^
	Shoot dry weight (g)	0.16 ± 0.02^b^	0.07 ± 0.02^c^	0.19 ± 0.02^a^	0.21 ± 0.03^a^	0.21 ± 0.03^a^
	Root dry weight (g)	0.04 ± 0.01^c^	0.01 ± 0.01^d^	0.04 ± 0.01b^c^	0.05 ± 0.01^a^	0.05 ± 0.01^ab^
	Socioeconomic mobility index	0.08 ± 0.01^b^	0.03 ± 0.01^c^	0.08 ± 0.02^b^	0.1 ± 0.01^a^	0.1 ± 0.02^a^
40 dpi	Shoot height (cm)	23.78 ± 1.81^c^	9.35 ± 0.27^e^	15.52 ± 0.25^d^	27.43 ± 0.5^b^	28.79 ± 1.01^a^
	Stem thickness (mm)	4.57 ± 0.17^bc^	4.2 ± 0.48^c^	4.44 ± 0.31^c^	4.92 ± 0.31^ab^	5.05 ± 0.24^a^
	Root length (cm)	22.26 ± 2.26^c^	15.64 ± 0.2^e^	18.49 ± 0.36^d^	23.63 ± 1.12^ab^	24.55 ± 2.49^a^
	Leaf area 1 (cm^2^)	41.56 ± 3.43^c^	23.54 ± 0.31^d^	38.69 ± 0.54^c^	68.5 ± 1.69^b^	73.43 ± 3.9^a^
	Leaf area 2 (cm^2^)	59.33 ± 7.18^b^	25.49 ± 0.39^d^	42.84 ± 0.53^c^	78.01 ± 1.71^a^	77.82 ± 10.82^a^
	Leaf area 3 (cm^2^)	88.08 ± 11.51^c^	10.42 ± 0.27^e^	68.44 ± 0.37^d^	101.62 ± 3.73^b^	121.2 ± 19.55^a^
	Leaf area 4 (cm^2^)	/	/	/	56.6 ± 1.42^b^	65.5 ± 4.71^a^
	Chlorophyll content (SPAD)	32.53 ± 5.57^b^	10.41 ± 0.24^c^	30.64 ± 0.41^b^	37.65 ± 1.12^a^	39.41 ± 2.56^a^
	Shoot fresh weight (g)	6.43 ± 0.88^c^	4.38 ± 0.23^d^	8.04 ± 0.51^b^	9.74 ± 0.2^a^	10.61 ± 1.34^a^
	Root fresh weight (g)	1.27 ± 0.33^b^	0.56 ± 0.19^c^	1.3 ± 0.25^b^	1.93 ± 0.18^a^	2.06 ± 0.1^a^
	Shoot dry weight (g)	0.37 ± 0.03^b^	0.14 ± 0.04^d^	0.31 ± 0.03^c^	0.52 ± 0.02^a^	0.63 ± 0.08^a^
	Root dry weight (g)	0.06 ± 0.01^b^	0.02 ± 0.01^c^	0.05 ± 0.01^b^	0.08 ± 0.01^a^	0.09 ± 0.02^a^
	Socioeconomic mobility index	0.07 ± 0.02^b^	0.03 ± 0.01^c^	0.07 ± 0.01^b^	0.11 ± 0.01^a^	0.11 ± 0.02^a^

a The “/” in the data indicates that the leaf area of the fourth true leaf is less than 5 cm^2^. Within the same column, different letters (a-e) indicate significant differences at *p* < 0.05 level.

**Table 3:** The incidence rate, disease severity index, and control efficacy of *B. velezensis* VJH504 against cucumber wilt disease.

Treatment	Disease incidence (%)	Disease index	Control efficacy (%)
*Foc*	96.21 ± 0.36^a^	68.80 ± 0.51^a^	
*Foc* + 0.1% Hymexazol	27.68 ± 0.11^b^	16.13 ± 0.22^b^	64.10 ± 0.06^a^
*Foc* + VJH504	19.01 ± 0.37^c^	9.25 ± 0.04^c^	81.76 ± 0.14^b^

The data are presented as mean ± standard deviation (SD). Within the same column, different letters (a-c) indicate significant differences at *p* < 0.05 level.

### 3.2. Effects of *Bacillus velezensis* VJH504 strain on the activities of defense-related enzymes in cucumber seedlings

At 20 dpi, the activities of LOX, PAL, CAT, PPO, and SOD in cucumber seedlings treated with *B. velezensis* VJH504 were significantly increased compared to the control treatment at a rate of 48.75, 48.68, 24.28, 227.83, and 50.49%, respectively. Significantly increased trends were also detected when the seedlings challenged with *Foc* at rates of 236.03, 81.64, 43.84, 223.98, and 51.67% in LOX, PAL, CAT, PPO, and SOD, respectively. It was observed that the activities of all enzymes in the seedlings inoculated with *Foc* after *B. velezensis* VJH504 treatment were significantly higher than those in the seedlings with *Foc* alone. However, PAL and CAT activities significantly increased after inoculation with *Foc* and treatment with fungicide in comparison to seeding inoculated with *Foc* alone, LOX, PPO, and SOD dramatically decreased (Table 4).

**Table 4:** Activities of five defense enzymes in cucumber leaves under different treatments.

	LOX(U/g)	PAL(U/g)	CAT(U/g)	PPO(U/g)	SOD(U/g)
CK	459.85 ± 33.31^d^	11.71 ± 1.13^e^	106.08 ± 3.78^e^	22.85 ± 1.61^d^	105.21 ± 4.06^d^
*Foc*	1545.24 ± 42.43^b^	21.27 ± 2.52^c^	152.59 ± 2.75^c^	74.03 ± 4.5^b^	159.57 ± 1.46^c^
*Foc* + 0.1% Hymexazol	636.59 ± 18.05^c^	28.18 ± 0.88^b^	184.74 ± 7.32^b^	43.76 ± 2.62^c^	195.91 ± 11.01^b^
*Foc* + VJH504	3924.7 ± 64.96^a^	34.91 ± 3.61^a^	255.84 ± 11.12^a^	193.19 ± 7.69^a^	231.74 ± 3.54^a^
VJH504	684.01 ± 13.76^c^	17.41 ± 1.1^d^	131.84 ± 3.35^d^	74.91 ± 3.4^b^	158.33 ± 2.88^c^

The data are presented as mean ± standard deviation (SD). Within the same column, different letters (a-e) indicate significant differences at *p* < 0.05 level.

### 3.3. Detection of antifungal and growth-promoting properties *in vitro*

**Figure 2:**
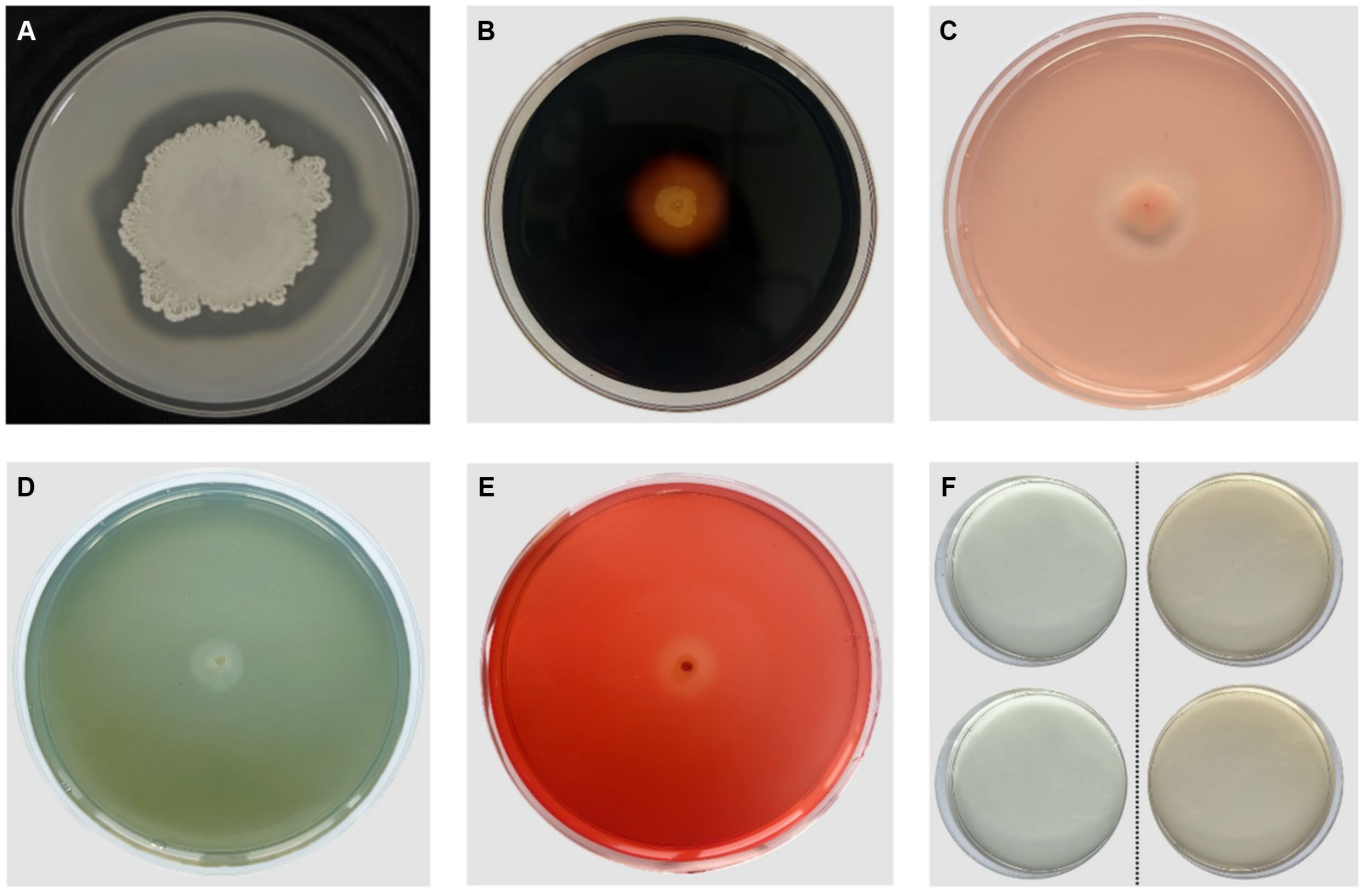
Antifungal and PGP (Plant Growth-Promoting) characteristics of *B. velezensis* VJH504. **(A)** Production of proteases; **(B)** Production of amylases; **(C)**Production of β-1,3-glucanases; **(D)** Production of iron carriers; **(E)** Production of cellulases; **(F)** Production of IAA. The two colorless transparent plates on the left side represent the control group without *B. velezensis* VJH504 inoculation, and the two orange transparent plates on the right side represent the treatment group with *B. velezensis* VJH504 inoculation.

*In vitro* antifungal and growth-promoting characteristic tests of *B. velezensis* VJH504 revealed that it secretes proteases, amylases, β-1,3-glucanases, and cellulases, which can degrade the cell walls of fungal pathogens in terms of antifungal properties. Additionally, it can produce siderophores and indole-3-acetic acid to promote plant growth (Figure 2).

### 3.4. The germination and growth promoting activity of *Bacillus velezensis* VJH504

Cucumber seeds treated with *B. velezensis* VJH504 exhibited a 5% higher germination rate and the average sprouts length than the water-treated ones (Supplementary Figure S1 and Supplementary Table S1).

**Figure 3:**
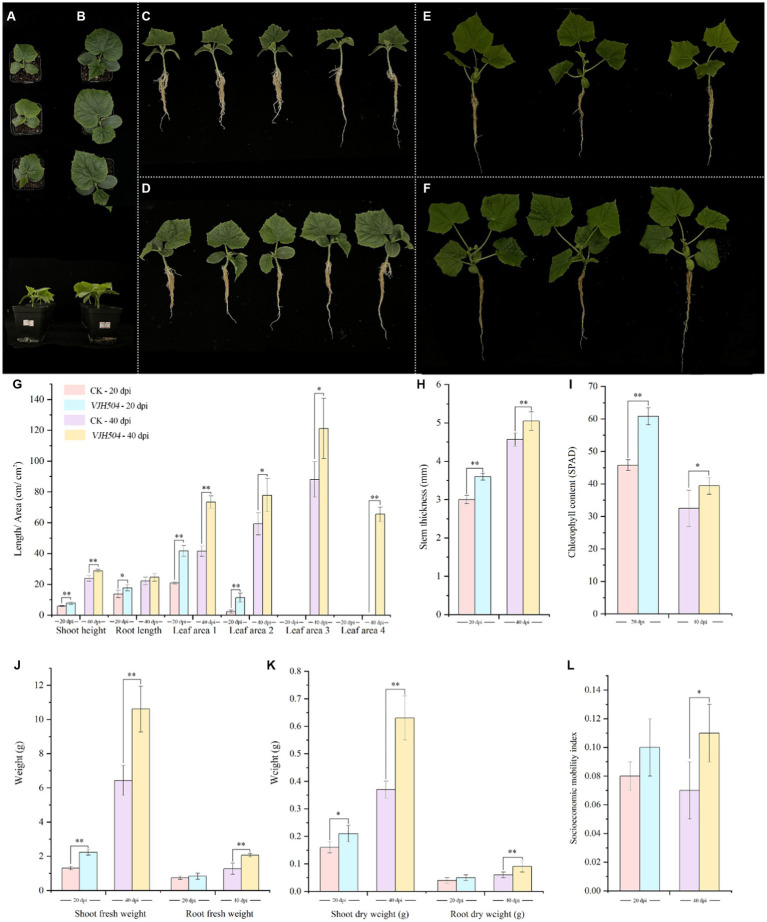
Effects of *B. velezensis* VJH504 on the promotion of cucumber seedling growth. **(A,C)** Cucumber seedlings treated with water at 20 dpi; **(B,D)** Cucumber seedlings inoculated with strain *B. velezensis* VJH504 at 20 days; **(E)** Cucumber seedlings treated with water at 40 dpi; **(F)** Cucumber seedlings inoculated with strain *B. velezensis* VJH504 at 40 dpi; **(G)** The plant height, root length and leaf area histogram of cucumber seedlings at 20dpi and 40dpi after CK and VJH504 treatment; **(H)** Stem diameter histogram of cucumber seedlings at 20dpi and 40dpi after CK and VJH504 treatment; **(I)** The chlorophyll content histogram of cucumber seedlings at 20dpi and 40dpi after CK and VJH504 treatment; **(J)** Shoot fresh weight and dry weight histogram of cucumber seedlings at 20dpi and 40dpi after CK and VJH504 treatment; **(K)** The underground fresh weight and dry weight histogram of cucumber seedlings at 20dpi and 40dpi after CK and VJH504 treatment; **(L)** Socioeconomic mobility index histogram of cucumber seedlings at 20dpi and 40dpi after CK and VJH504 treatment.

At 20 and 40 days post inoculation (dpi), the plant height, stem diameter, and aboveground fresh weight of *B. velezensis* VJH504 increased considerably (Figures 3G–L). At 20 dpi, the leaf area treated with *B. velezensis* VJH504 likewise increased significantly at 20dpi (Figure 3G). In comparison to the control, the first and second true leaves’ areas grew by, respectively, by 99.16 and 385.1%. Then, the average leaf area of cucumber seedlings treated with *B. velezensis* VJH504 reached 65.5 cm^2^ by fourth true leaf, but it was less than 5 cm^2^ from the control treatment at 40 dpi, which was the most notable difference between the two treatments (Figure 3G). At the same time, after 40 days of inoculation, the fresh weight and dry weight of the aboveground part of cucumber seedlings and the fresh weight and dry weight of the underground part were significantly higher than those of the control group (Figures 3J,K). The socioeconomic mobility index of VJH504 treatment (0.115) was significantly higher than that of the control (0.073) at 40 dpi (Figure 3L).

### 3.5. Whole genome sequencing and assembly of *Bacillus velezensis* VJH504

**Figure 4:**
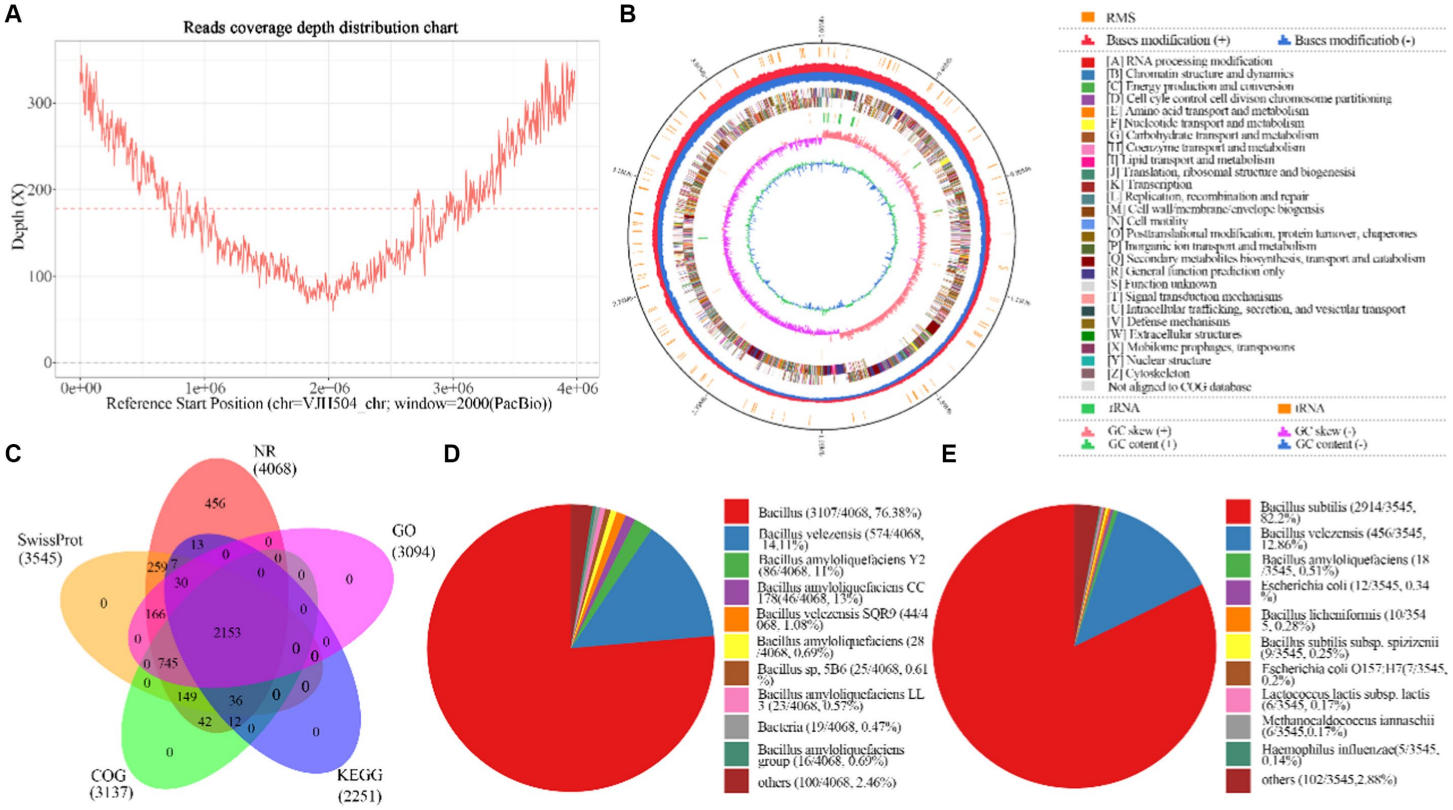
Basic information about the whole genome sequencing and assembly of *B.velezensis VJH504*. **(A)** Assembly of genome coverage depth distribution plot; **(B)** Genomic map of *B. velezensis VJH504*. From the inside out, the distribution of the circles is as follows: the first circle represents the genome size (black line); the second circle represents the distribution of the restriction modification systems on the forward (red) and reverse (blue) strands; the third circle represents the COG classification of protein-coding genes, located on both the forward and reverse strands; the fourth circle represents the distribution of tRNA (brown) and rRNA (green); the fifth circle represents the GC skewness; the sixth circle represents the GC content; **(C)** Analysis and statistical plot of shared and unique annotations in the base database; **(D)** Top 10 species distribution plot in NR library. Different colored sectors represent the proportion of annotations to each species, while “others” represents the collection of species outside the top 10; **(E)** Top 10 species distribution plot in SwissProt database. Different colored sectors represent the proportion of annotations to each species, while “others” represents the collection of species outside the top 10.

In this study, PacBio RSII sequencing technology combined with NGS sequencing technology was used to construct two sequencing libraries, including second-generation and third-generation sequencing, to efficiently and accurately complete the whole genome sequencing of the strain and investigate its disease resistance mechanisms. A total of 65,420 High Fidelity reads were generated from DNA sequencing of the strain, with an average length of 10,865.54 bp and an N50 of 11,273 bp. The average genome coverage was 178.41X (Figure 4A). The assembled genome sequence has been deposited in GenBank (Accession No. CP131928). The strain’s genome consists of a circular chromosome with a size of 3,980,733 bp, and a GC content of 46.46%. Eight CRISPR structures, three genomic islands, two prophages, 149 tandem repeat sequences (TRF), and nine microsatellite DNA sequences (SSR) were detected (Supplementary Table S2). A total of 4,104 protein-coding genes were predicted, covering 89.21% of the genome, while the remaining 195 genes were identified as tRNA (86 genes), rRNA (27 genes), and other non-coding RNA (82 genes) (Figure 4B). The predicted protein sequences of the genes were annotated against corresponding databases (E-value = 1e-5), and 4,068 (99.12%), 3,545 (86.38%), 3,137 (76.44%), 2,251 (54.85%), and 3,094 (75.39%) protein-coding genes showed significant matches in the NR, SwissProt, COG, KEGG, and GO databases, respectively (Figure 4C). In the NR database, 76.38% of the sequences showed the highest similarity to sequences from Bacillus species within the genus. The remaining sequences had significant similarity mainly with *B. velezensis* (14.11%) sequences, followed by *B. amyloliquefaciens* Y2 (2.11%) and *B. amyloliquefaciens* CC178 (1.08%) (Figure 4D). In the SwissProt database, 82.2% of the sequences showed the highest similarity to *B. subtilis* sequences, followed by *B. velezensis* (12.86%) and *B. amyloliquefaciens* (0.51%) (Figure 4E).

### 3.6. Species identification and phylogenetic analysis of *Bacillus velezensis* VJH504 strain

**Figure 5:**
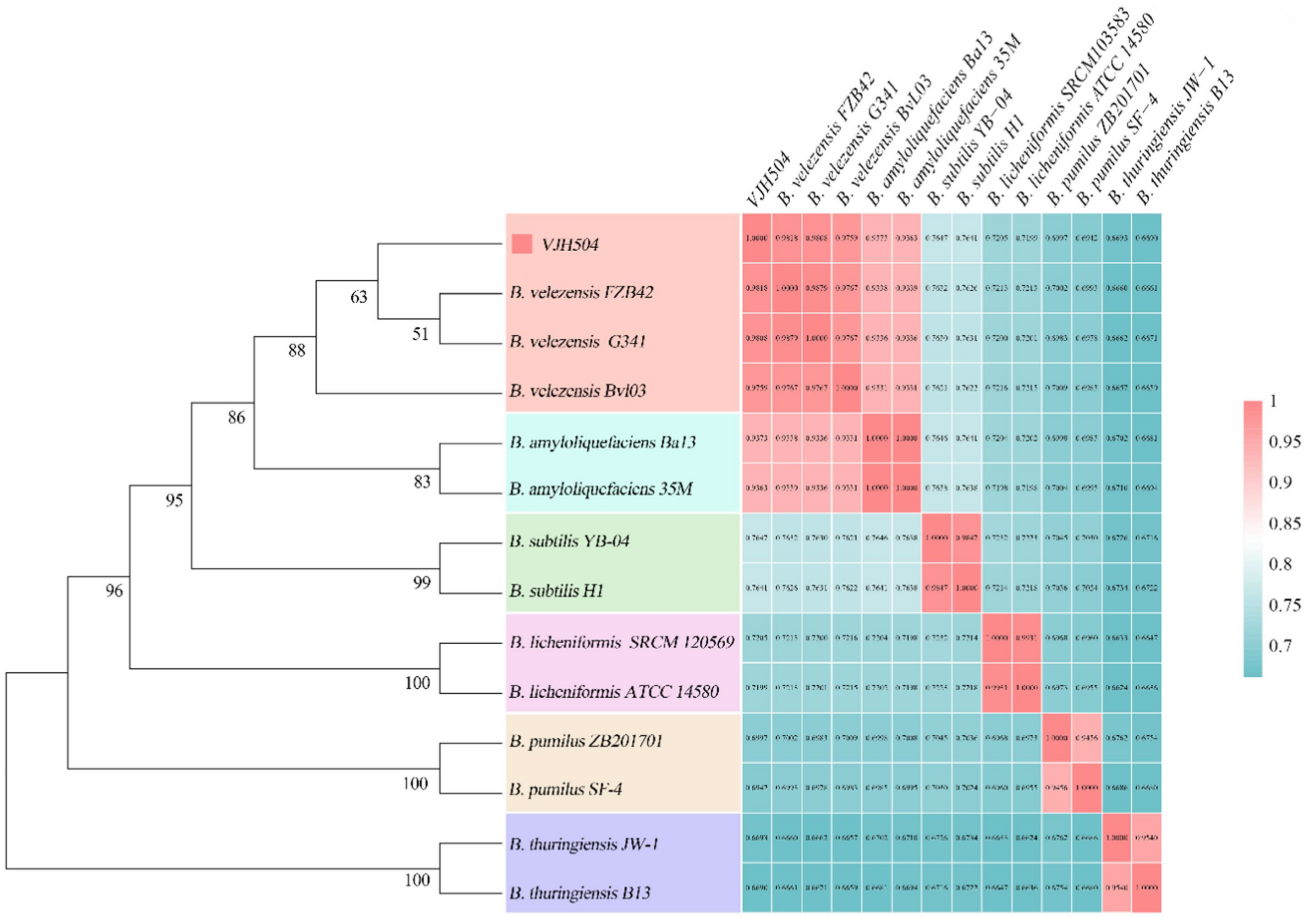
Species identification of *B. velezensis* VJH504. The left half represents the phylogenetic tree of *B. velezensis* VJH504 and 13 other species of the genus Bacillus based on the 16S rRNA sequence. The right half represents the ANI (Average Nucleotide Identity) analysis of *B. velezensis* VJH504 and other 13 species of Bacillus.

A phylogenetic tree was constructed based on the 16S rRNA gene sequences of *B. velezensis* VJH504 and 13 other Bacillus strains (Figure 5). *B. velezensis* VJH504 clustered together with three strains of *B. velezensis*, and its closest relatives were *B. velezensis* FZB42 and *B. velezensis* G341. Whole-genome comparisons between *B. velezensis* VJH504 and other strains revealed that the highest average nucleotide identity (ANI) values (97% and above) were only observed with *B. velezensis* strains (Figure 5). The genome matches with *B. velezensis* FZB42, *B. velezensis* G341, and *B. velezensis* BvL03 were 98.12, 98.05, and 97.55%, respectively, which were above the threshold range of 95 to 96% for bacterial species. *B. velezensis* VJH504 showed the highest ANI value with *B. velezensis* FZB42, consistent with the clustering observed in the 16S rRNA tree. In contrast, the ANI values between *B. velezensis* VJH504 and the other 13 Bacillus strains were much lower, ranging from 66.53 to 72.03%. Therefore, based on the comparison of the 16S rRNA and whole-genome sequences, *B. velezensis* VJH504 should be classified as *B. velezensis*.

### 3.7. Genome-specific functional annotation of *Bacillus velezensis* VJH504

After analysis against the CARD database in this study, a total of 329 antibiotic resistance genes were identified in *B. velezensis* VJH504, including those involved in antibiotic synthesis and antibiotic resistance (Supplementary Table S3).

After annotation against the VFDB database, a total of 693 virulence factor genes were identified in *B. velezensis* VJH504, including the quorum sensing regulator gene *luxS* (Supplementary Table S4).

**Figure 6:**
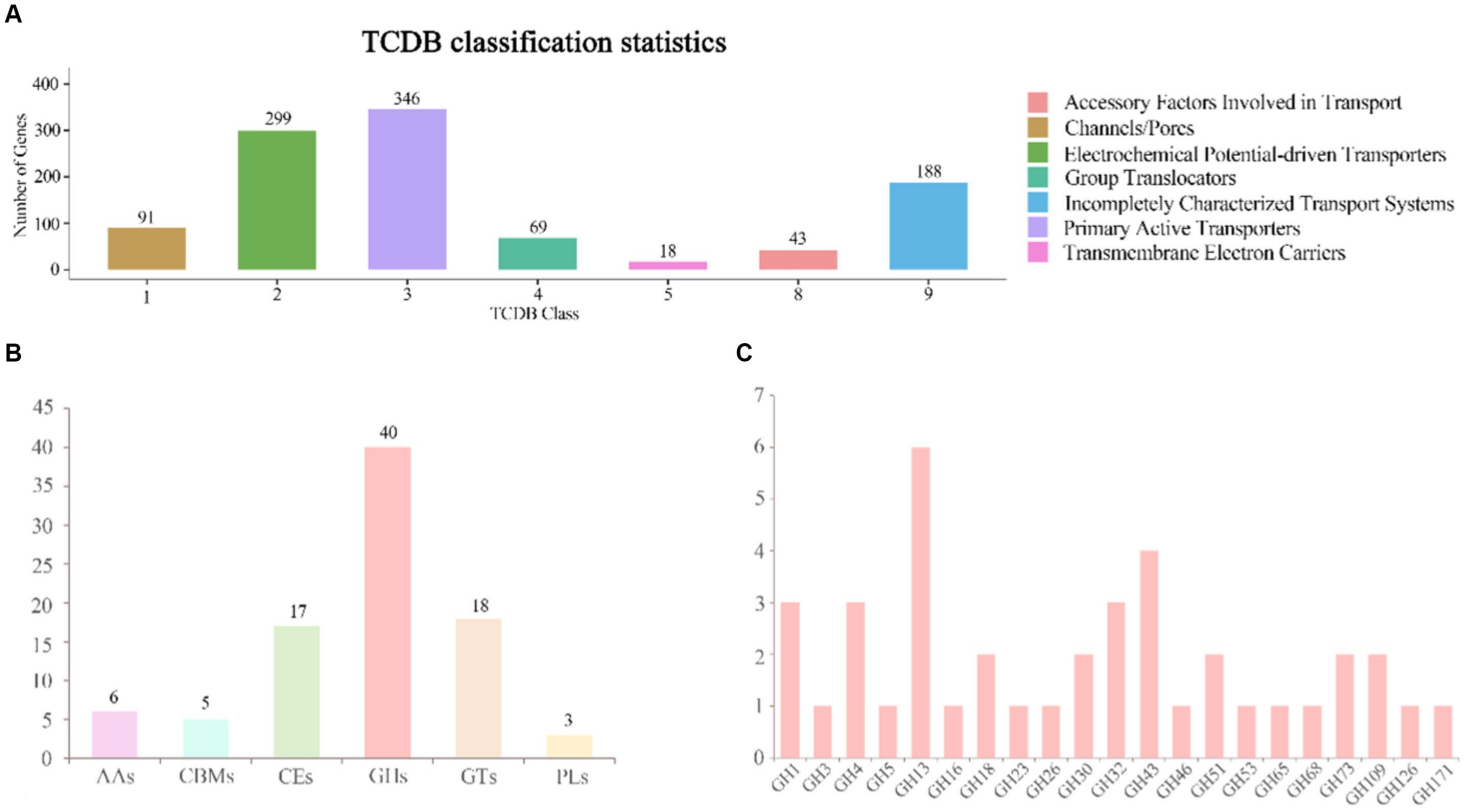
Annotation of the TCDB database and distribution of CAZy families in the genome of *B. velezensis* VJH504. **(A)** TCDB classification statistical graph. The horizontal axis represents transporter classes, and the vertical axis represents the number of genes annotated to each corresponding class, while the legend corresponds to the definition of each transporter class. **(B)** Gene count distributions of carbohydrate-active enzyme (CAZy) families in *B. velezensis* VJH504 genome; **(C)** Functional characterization of glycoside hydrolase family is based on the CAZy database.

In *B. velezensis* VJH504 genome, a total of 1,054 genes were annotated in the TCDB database. These genes encoded for proteins involved as Primary Active Transporters (246), Electrochemical Potential-driven Transporters (299), Incompletely Characterized Transport Systems (188), Channel/Pores (91), Group Translocators (69), Accessory Factors Involved in Transport (43), and Transmembrane Electron Carriers (18) (Figure 6A).

### 3.8. Genome CAZymes analysis of *Bacillus velezensis* VJH504

The genomic analysis of selected genes in *B. velezensis* VJH504 identified 86 genes as putative CAZymes, including 40 Glycoside Hydrolases (GHs), 18 Glycosyl Transferases (GTs), 17 Carbohydrate Esterases (CEs), 6 Auxiliary Activities (AAs), 3 Polysaccharide Lyases (PLs), and 5 Carbohydrate-Binding Modules (CBMs), which play a crucial role in facilitating the binding of enzymes to their substrates. Among them, three genes, orf01880, orf01885, and orf03428, were classified as both CHs and CBMs (Figure 6B). Besides, all CAZymes possessed N-terminal signal peptides, which are involved in the secretion of proteins across the cytoplasmic membrane, indicating that they are secreted enzymes. The antifungal potential of CAZymes in the GH family, such as β-glucanases (GH1), β-glucosidases (GH3), cellulases (GH5), trehalases (GH68), endo-glucanases (GH5, GH51), chitinases (GH18), and starch branching enzymes (GH13_31, GH13_28, GH13_29), suggests their ability to inhibit the growth of plant pathogens. The distribution of CAZymes in *B. velezensis* VJH504 genome indicates its significant capacity to antagonize both bacteria and fungi (Figure 6C).

### 3.9. Secondary metabolic potential of *Bacillus velezensis* VJH504

Within the genome of *B. velezensis* VJH504, 12 gene clusters related to the biosynthesis of secondary metabolites were identified (Table 5). These clusters included four clusters encoding NRPS (Non-Ribosomal Peptide Synthetases), three clusters encoding transAT-PKS (trans-Acyltransferase Polyketide Synthases), two clusters involved in terpene biosynthesis, one cluster of T3PKS (Type III Polyketide Synthases), one cluster of PKS-like (Type III Polyketide-like Synthases), one cluster involved in lantipeptide biosynthesis, and one cluster highly similar to a gene cluster associated with bacteriocin synthesis. Among the three NRPS-encoding clusters, two clusters exhibited 100% similarity to known fengycin and bacillibactin synthesis clusters, while the other cluster showed 82% similarity to a known surfactin synthesis cluster. The three transAT PKS-encoding clusters exhibited 100% similarity to clusters involved in the synthesis of macrolactin H, bacillaene, and difficidin, respectively. Four biosynthetic gene clusters with no similarity in the antiSMASH database were also found, with two clusters encoding terpene biosynthesis and the other two clusters encoding lanthipeptide-class-ii and T3PKS, respectively. These clusters represent potential novel bioactive compound biosynthetic gene clusters that require further isolation and identification. Based on their matches with the antiSMASH database, two of these clusters were involved in terpene biosynthesis, while the remaining clusters were associated with lanthipeptide-class-ii and T3PKS biosynthesis. The genome of *B. velezensis* FZB42 contains 13 gene clusters related to the synthesis of secondary metabolites, including four clusters encoding NRPS, four clusters encoding transAT-PKS, two clusters of terpene biosynthesis, etc.; twelve gene clusters related to secondary metabolite synthesis were predicted in the genome of *B. velezensis* G341, including four clusters encoding NRPS, four clusters encoding transAT-PKS, two clusters encoding terpene biosynthesis, and one cluster encoding lanthipeptide-class-ii. The genome of *B. velezensis* BvL03 contains 14 gene clusters related to the synthesis of secondary metabolites, including five gene clusters encoding NRPS, four gene clusters encoding transAT-PKS, one gene cluster encoding terpene biosynthesis, and one gene cluster encoding lanthipeptide-class-ii. All 12 gene clusters encoding secondary metabolites in *B. velezensis* VJH504 genome were also present in the genomes of three other *B. velezensis* strains (Supplementary Table S5).

**Table 5:** The hypothetical gene cluster lists encoding secondary metabolites predicted by antiSMASH in the genomes of *B. velezensis* VJH504.

Clusters	Types	Start	End	Most similar known clusters	Similarity
Clusters 1	NRPS	323,410	387,387	surfactin	82%
Clusters 2	PKS-like	924,057	965,301	butirosin A/butirosin B	7%
Clusters 3	terpene	1,050,180	1,067,588		
Clusters 4	lanthipeptide-class-ii	1,188,578	1,217,466		
Clusters 5	transAT-PKS	1,384,086	1,471,921	macrolactin H	100%
Clusters 6	transAT-PKS, T3PKS, NRPS	1,691,450	1,792,015	bacillaene	100%
Clusters 7	NRPS, transAT-PKS, betalactone	1,865,757	2,000,067	fengycin	100%
Clusters 8	terpene	2,028,705	2,050,588		
Clusters 9	T3PKS	2,113,906	2,155,006		
Clusters 10	transAT-PKS	2,282,382	2,376,174	difficidin	100%
Clusters 11	NRP-metallophore, NRPS, RiPP-like	3,051,859	3,103,650	bacillibactin	100%
Clusters 12	other	3,639,960	3,681,378	bacilysin	100%

### 3.10. Genome comparison

**Figure 7:**
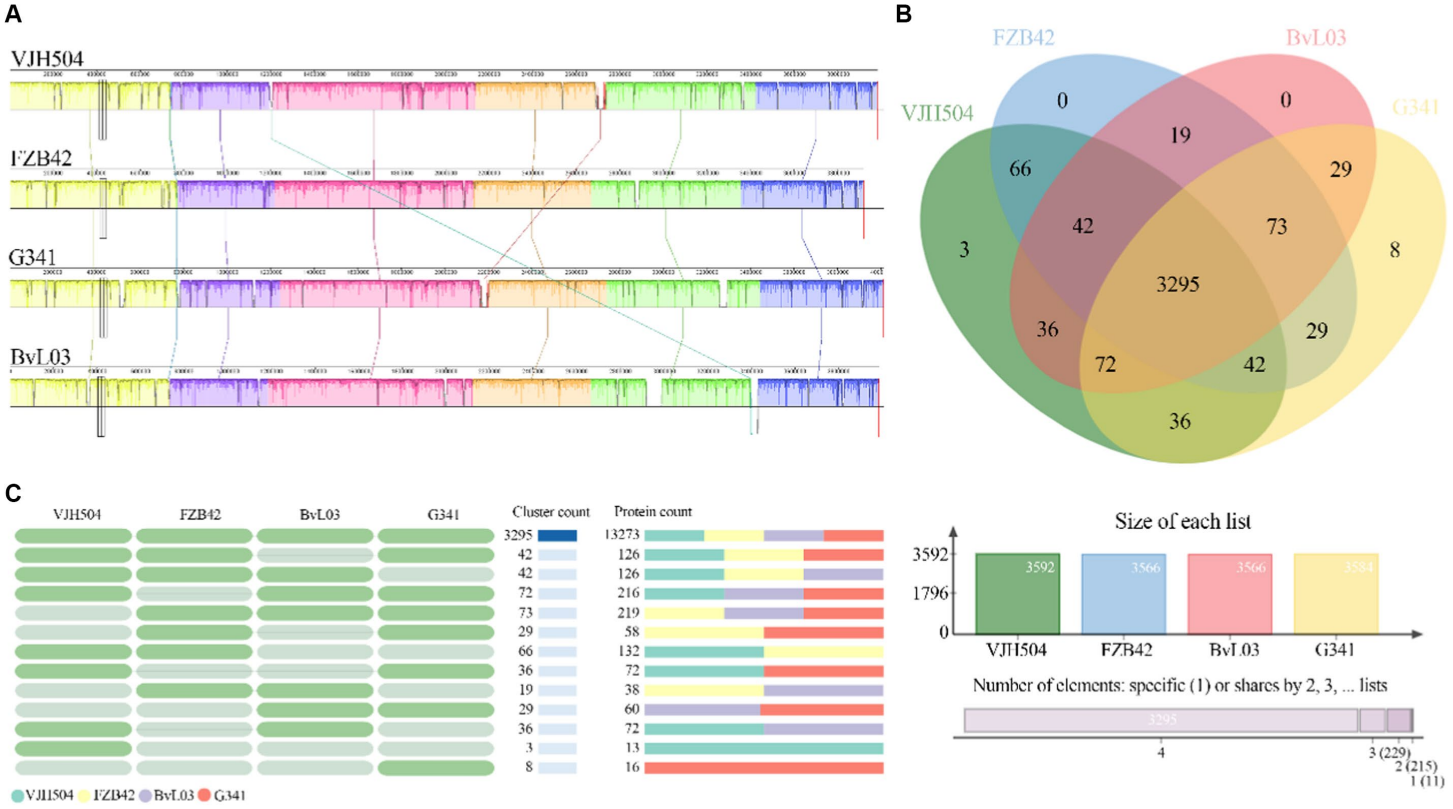
Comparative genomic analysis. **(A)** Collinearity analysis of *B. velezensis* VJH504 with three other *B. velezensis* strains. **(B)** Venn diagram showing the number of unique and shared gene clusters between *B. velezensis* VJH504 and three other *B. velezensis* strains. **(C)** Presence or absence of orthologous gene clusters in different species. Green indicates presence, gray indicates absence.

To perform a syntenic analysis of *B. velezensis* VJH504 strain’s genome with other *B. velezensis* strains, the full genome sequences from the NCBI sequence database (FZB42, G341, and BvL03) were analyzed for syntenic regions. This was accomplished by using the Mauve software, which effectively identifies conserved genomic regions in multiple genomes, rearrangements, and more. The results show that the three *B. velezensis* strains and *B. velezensis* VJH504, share similar genome structures and have no genetic mutations. However, *B. velezensis* VJH504 and G341 as well as BvL03 have a minor genetic inversion (Figure 7A).

Furthermore, a pan-genomic analysis of the whole genomes was conducted to compare *B. velezensis* VJH504 with *B. velezensis* FZB42, *B. velezensis* G341, and *B. velezensis* BvL03. As shown in the Figure 7B, the number of clusters in *B. velezensis* VJH504, *B. velezensis* FZB42, *B. velezensis* G341, and *B. velezensis* BvL03 are 3,592, 3,566, 3,584, and 3,566, respectively. The number of singletons is 479, 120, 156, and 177, respectively (Supplementary Table S6). The number of shared homologous genes among the four strains is 3,295. *B. velezensis* VJH504 possesses three unique homologous gene clusters, encoding a total of 13 proteins. *B. velezensis* G341 has eight unique homologous gene clusters, encoding 16 proteins. However, *B. velezensis* FZB42 and *B. velezensis* BvL03 do not have any unique homologous gene clusters (Figure 7C).

## 4. Discussion

Perior studies have noticed the major constraint on the quality and yield of cucumber production due to Cucumber wilt disease. Thus, the safe and effective control of cucumber wilt disease has become a hot topic in recent years. Microbial biocontrol agents have gained increasing attention due to their effectiveness, environmental friendliness, and safety for humans and livestock (Berg, 2009). As an important biocontrol agent, *B. velezensis* is a relatively novel species, and research on the biocontrol mechanisms of this species is gradually increasing (Liu et al., 2017; Martinez-Raudales et al., 2017; Dong et al., 2023; Li et al., 2023). Previous reports on the complete genome of *B. velezensis* mainly focused on the general characteristics of the genome and its effects on growth promotion, with less emphasis on in-depth analysis of biocontrol functions and mechanisms. The present study was designed to expand our understanding of the molecular biocontrol mechanisms of *B. velezensis* VJH504, *in vivo* and vitro biocontrol experiments as well as growth promotion experiments, confirming its excellent growth-promoting and biocontrol effects. Therefore, the complete genome of *B. velezensis* VJH504 was sequenced, and the strain consists of a 3,980,733 bp circular chromosome with a GC content of 46.46%. Subsequently, its genome was compared with other Bacillus strains.

Thereafter, the phylogenetic tree analysis using the 16S rRNA gene sequences was carried out, and found that the VJH504 strain clusters were closely with *B. velezensis* FZB42, *B. velezensis* G341, and *B. velezensis* BvL03, indicating its close relationship with these *B. velezensis* strains. The gene relatedness of VJH504 was further analyzed based on Average Nucleotide Identity (ANI). It is apparent that the ANI values between VJH504 and all *B. velezensis* strains were above 97%, surpassing the threshold of 96%. Strong suggestion of a higher degree of genomic homogeneity between VJH504 and *B. velezensis* FZB42 due to the results showed that the highest ANI value of VJH504 with *B. velezensis* FZB42. Moreover, the comparative genomic analysis revealed that VJH504 strain has the highest number of homologous genes and single-copy genes among the strains examined. The most striking results that VJH504 strain possesses certain specificity, and its biocontrol mechanisms exhibit some differences compared to other *B. velezensis* strains.

Turning to the conditions of soil microecology, microorganisms compete intensively due to limited available resources, mainly in terms of living space and nutrient competition. Previous studies proved that the formation of biofilms is an important characteristic related to the colonization ability of biocontrol microorganisms (Ongena and Jacques, 2008), which enables beneficial bacteria to compete for ecological niches with pathogenic bacteria. Later, Chen et al. (2012) studies revealed that *B. subtilis* 3,610 formed biofilms on tomato roots, enhancing its colonization ability in the biocontrol process against tomato bacterial wilt. Futhermore it was discovered that the formation ability of biofilms positively correlated with biocontrol efficacy against *Ralstonia solanacearum*, which is the causal agent of bacterial wilt, by the construction and evaluation of biofilm deficient and enhanced mutants. It has been reported that quorum sensing significantly affects biofilm formation in bacteria (Xu et al., 2013). Our observation identified an important quorum sensing regulator gene *luxS* in *B. velezensis* VJH504., which is a key regulatory gene involved in quorum sensing mediated by autoinducer-2 (AI-2) (Federle, 2009).The exciting results of Xiong et al. (2020) showed that AI-2 positively affected the colonization of *B. velezensis* SQR9 on plant roots. According to the aforementioned findings, *B. velezensis* VJH504 may utilize biofilm formation as a strategy for disease control.

On the other hand, other Bacillus isolates, such as *B. subtilis*, *B. amyloliquefaciens*, and *B. velezensis*, have been found to inhibit grasses’ spore germination or hyphal growth. In dual cultures of plant pathogens and antagonistic bacteria, the hyphae of the pathogenic fungi exhibit deformities. Ferreira et al. (1991) isolated *B. subtilis* from grapevine wood, which could inhibit the growth of the grapevine trunk disease pathogen *Eutypa lata* and induce abnormal hyphal growth. When the concentration of *B. subtilis* was equal to or greater than 0.8 mg/mL, its antibiotic substances completely inhibited the germination of *Eutypa lata* spores. Zhang et al. (2020b) discovered that *B. subtilis* ZD01, which exhibited strong antifungal activity, produced volatile organic compounds that caused significant morphological changes in *Alternaria solani*, inhibiting its conidial germination and reducing the lesion area and pathogen population. The findings of this study mirror those of the previous studies that have examined the morphological changes in fungi. In our case, the microscopic images showed that *B. velezensis* VJH504 could inhibit the hyphal development of *Foc* and induce abnormal swelling of spores and hyphae in dual-culture antagonism tests. Additionally, the genome annotation of *B. velezensis* VJH504 revealed a total of 329 antibiotic resistance genes with a large number of CAZymes genes, indicating its potential for degradation and utilization of fungal polymers as a nutrient source. Its strong capability in combating fungal pathogenswas proved previously (Banani et al., 2015; Chen et al., 2018). The abnormal hyphal growth of *Foc* induced by *B. velezensis* VJH504 may be caused by the production of enzymes, detected in the culture supernatant, which degrade chitin, glucans, and other polymers in the fungal cell wall. This mechanism has been reported in many Bacillus species, including Naglot et al. (2015), who found that *B. circulans* SDRLIN-1 produced cellulase, β-1,3-glucanase, pectinase, amylase, protease, and chitinase, which led to the swelling and deformation of fungal pathogen hyphae. Chitin-degrading enzymes also produced by *B. cereus* and *Enterobacter agglomerans* have been used for the biocontrol of *R. solani* (Pleban et al., 1997).

Sequencing of the genome of *B. velezensis* VJH504 has revealed the presence of gene clusters encoding several secondary metabolites. Among the observed compounds, previous reports have shown that terpenes (Gershenzon and Dudareva, 2007), lanthipeptides (McAuliffe et al., 2001), bacilysin (Wu L. M. et al., 2015), fengycin (Hanif et al., 2019), bacillibactin (Arguelles-Arias et al., 2009), bacillaene (Arguelles-Arias et al., 2009) and macrolactin H (Ortiz and Sansinenea, 2020) exhibit inhibitory activity against filamentous fungi. Reporting previous researches for explaining the mechanisms of secondary metabolites, they have shown that certain terpenes can inhibit the germination process of fungal spores by influence spore germination and mycelial growth through multiple mechanisms (Akino et al., 1995; De la Cruz-Lopez et al., 2022). As for Fengycin, it showed inhibition the growth of fungal hyphae (Wu et al., 2021). Similarly, Lanthipeptides, produced by *B. velezensis* including species of *Foc*, possess antibacterial activity against several plant pathogen (McAuliffe et al., 2001; Holtsmark et al., 2006; Palazzini et al., 2016; Zhao et al., 2021).

Regarding Bacilysin, it is the major contributor to the antagonistic of *B. velezensis* against gram-negative foodborne pathogens and exhibits broad-spectrum antibacterial activity against both bacteria and fungi (Nannan et al., 2021). By causing structural damage to the fungal cell wall, leading to cell lysis and death, thus exerting strong fungicidal and inhibitory effects (Perry and Abraham, 1979; Loeffler et al., 1986). While the chelating agent Bacillibactin is a polyene antibiotic that antagonizes bacteria and fungi by inhibiting protein synthesis (Patel et al., 1995).It has high affinity for iron ions and competitively binds to soluble iron ions, which are necessary for the growth and activity of pathogenic bacteria in the environment (Hotta et al., 2010). Taking into account the previous studies and findings from current study, they support the idea of the potential antifungal secondary metabolites and the secretion capacity of potential antifungal CAZymes (carbohydrate-active enzymes) in *B. velezensis* VJH504 that it can significantly inhibit the growth of plant pathogens. It is worth noting that comparative genomic analysis of *B. velezensis* VJH504 and three other *B. velezensis* strains revealed that *B. velezensis* VJH504 has 3 unique gene clusters encoded a total of 13 different proteins. Where, four gene clusters did not have homologous proteins in the database, but one gene cluster showed similarity with only 7% to surfactin. One unanticipated result that there might be novel antimicrobial substances needs further work on *B. velezensis* VJH504.

In the matter of enhancing plant nutrient uptake growth-promoting capabilities using microorganisms, it can be supported by producing iron carriers. Most beneficial bacteria secrete low-molecular-weight iron chelators when iron is limited, improving microbial absorption and transport of iron ions and increasing iron ion mobility in the environment, thereby significantly increasing plant biomass production (Li et al., 2019). Like so, Bacillus can convert atmospheric N2 into nitrogen elements for plant uptake and utilization and synthesize auxins, cytokinins, and induce indole-3-acetic acid synthesis, thereby promoting plant growth (Muttilainen et al., 1995; He et al., 2002). In this study, the *in vitro* growth-promoting characteristics of *B. velezensis* VJH504 were evaluated andfound that *B. velezensis* VJH504 can produce iron carriers and indole-3-acetic acid. The growth-promoting experiments demonstrated that *B. velezensis* VJH504 has the ability to significantly enhance the growth of cucumber seedlings. Therefore, it can be assumed that *B. velezensis* VJH504 can effectively prevent cucumber wilt disease by promoting cucumber growth.

Ongena et al. (2002) first demonstrated that the lipopeptide antibiotics surfactin and fengycin produced by *B. subtilis* can trigger the plant defense response through the salicylic acid (SA) pathway and induce resistance mechanisms in soybeans. Subsequently, research on Bacillus-induced systemic resistance (ISR) in plants has gained significant attention. In this study, analysis of the secondary metabolite gene clusters in *B. velezensis* VJH504 revealed the presence of gene clusters encoding surfactin and fengycin. Therefore, it can be inferred that *B. velezensis* VJH504 has the potential to induce similar systemic resistance in plants.

**Figure 8:**
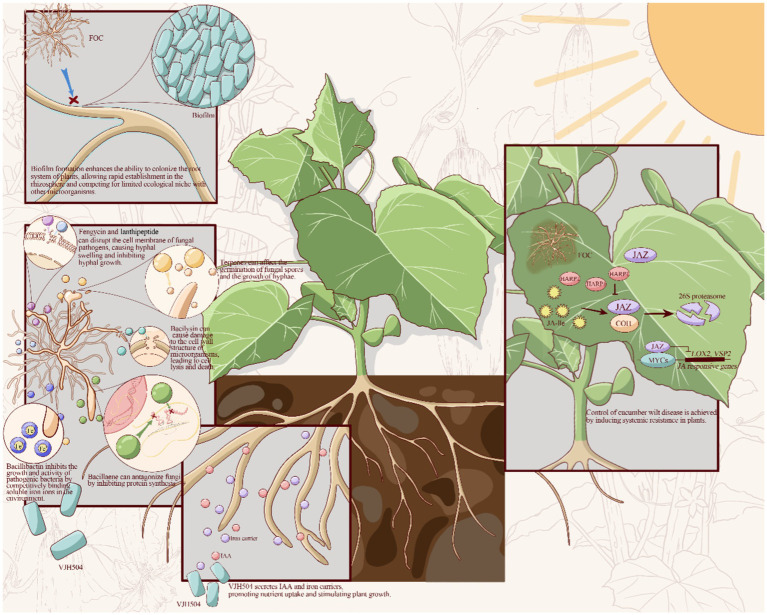
Prediction of the biocontrol mechanisms of *B. velezensis* VJH504.

This study set out to determine growth-promoting characteristic biocontrol capacity and the complete genome sequencing of the isolated *B. velezensis* VJH504. Based on the experimental and genomic analysis, particularly in the aspects of biofilm formation, antibiotic synthesis, promotion of plant growth, and production of systemic resistance-inducing agents, the biocontrol mechanisms of *B. velezensis* VJH504 can be inferred as follows: (1) the formation of Biofilm enhance colonization ability in the plant root system, enabling rapid establishment in the rhizosphere and occupying ecological niches where competition with *Foc* is limited; (2) Secretion of cell wall-degrading enzymes and secondary metabolites such as terpenes, lanthipeptides, bacilysin, fengycin, bacillibactin, bacillaene, and macrolactin H antagonize phytopathogenic fungi; (3) Production of iron carriers and indole-3-acetic acid promote nutrient uptake and plant growth, thereby achieving disease control; (4) Inducing systemic resistance in plants control cucumber wilt disease (Figure 8).

This is a bundant conclusion for further progress in analyzing, analyzing the regulatory network of functional genes through big data analysis, studying the interaction mechanisms of *B. velezensis* with host organisms in more depth. More research should also conduct the selection of suitable strains for specific ecological systems by designing different environments to enhance their effectiveness. Along with employing fluorescent tracking methods can be used to analyze the colonization ability of *B. velezensis* in plant roots, leaves, and animal intestines. Besides enhancing strains’ colonization ability and increasing their application value, Gene clusters related to colonization should be explored, and molecular genetic improvement techniques used Simultaneously, more attention should be paid to large-scale production and use with reasonable assessment of biosafety, and regulation to can be applied in the field.

## Data availability statement

The datasets presented in this study can be found in online repositories. The names of the repository/repositories and accession number(s) can be found at: https://www.ncbi.nlm.nih.gov/genbank/, CP131928.

## Author contributions

FY: Conceptualization, Investigation, Methodology, Writing – original draft. HJ: Data curation, Formal analysis, Software, Visualization, Writing – original draft. KM: Funding acquisition, Writing – review & editing. XW: Supervision, Writing – review & editing. SL: Funding acquisition, Supervision, Writing – review & editing. YC: Supervision, Writing – review & editing. YJ: Supervision, Writing – review & editing. BT: Supervision, Writing – review & editing. XS: Supervision, Writing – review & editing.

## References

[ref1] Afzal A. Bahader S. Ul Hassan T. Naz I. Din A. Z. (2023). Rock phosphate solubilization by plant growth-promoting *bacillus velezensis* and its impact on wheat growth and yield. Geomicrobiol J. 40, 131–142. doi: 10.1080/01490451.2022.2128113

[ref2] Akino T. Turushima T. Yamaoka R. (1995). 3-formyl-7,11-dimethyl-(2E, 6Z, 10)-dodecatrienal - antifungal compound in the mandibular gland of the ant, lasius-fuliginosus latreille. Nippon Nogeikagaku Kaishi 69, 1581–1586. doi: 10.1303/jjaez.39.329

[ref3] Al-Naamani L. Dobretsov S. Al-Sabahi J. Soussi B. (2015). Identification and characterization of two amylase producing bacteria Cellulosimicrobium sp. and Demequina sp. isolated from marine organisms. J. Agric. Mar. Sci. 19, 8–15. doi: 10.24200/jams.vol20iss0pp8-15

[ref4] Arguelles-Arias A. Ongena M. Halimi B. Lara Y. Brans A. Joris B.. (2009). *Bacillus amyloliquefaciens* GA1 as a source of potent antibiotics and other secondary metabolites for biocontrol of plant pathogens. Microb. Cell Factories 8:12. doi: 10.1186/1475-2859-8-63, PMID: 19941639 PMC2787494

[ref5] Banani H. Spadaro D. Zhang D. P. Matic S. Garibaldi A. Guilin M. L. (2015). Postharvest application of a novel chitinase cloned from Metschnikowia fructicola and overexpressed in Pichia pastoris to control brown rot of peaches. Int. J. Food Microbiol. 199, 54–61. doi: 10.1016/j.ijfoodmicro.2015.01.002, PMID: 25632799

[ref6] Bartholomew E. S. Xu S. Zhang Y. Q. Yin S. Feng Z. X. Chen S. Y.. (2022). A chitinase CsChi23 promoter polymorphism underlies cucumber resistance against fusarium oxysporum f. sp. cucumerinum. New Phytol. 236, 1471–1486. doi: 10.1111/nph.18463 36068958

[ref7] Berg G. (2009). Plant-microbe interactions promoting plant growth and health: perspectives for controlled use of microorganisms in agriculture. Appl. Microbiol. Biotechnol. 84, 11–18. doi: 10.1007/s00253-009-2092-7, PMID: 19568745

[ref8] Blin K. Shaw S. Steinke K. Villebro R. Ziemert N. Lee S. Y.. (2019). antiSMASH 5.0: updates to the secondary metabolite genome mining pipeline. Nucleic Acids Res. 47, W81–W87. doi: 10.1093/nar/gkz310, PMID: 31032519 PMC6602434

[ref9] Chen Y. Chai Y. R. Gu J. H. Losick R. (2012). Evidence for cyclic di-GMP-mediated signaling in *Bacillus subtilis*. J. Bacteriol. 194, 5080–5090. doi: 10.1128/jb.01092-12, PMID: 22821967 PMC3430322

[ref10] Chen P. H. Chen R. Y. Chou J. Y. (2018). Screening and evaluation of yeast antagonists for biological control of botrytis cinerea on strawberry fruits. Mycobiology 46, 33–46. doi: 10.1080/12298093.2018.1454013, PMID: 29998031 PMC6037076

[ref12] De la Cruz-Lopez N. Cruz-Lopez L. Holguin-Melendez F. Guillen-Navarro G. K. Huerta-Palacios G. (2022). Volatile organic compounds produced by cacao endophytic bacteria and their inhibitory activity on moniliophthora roreri. Curr. Microbiol. 79:35. doi: 10.1007/s00284-021-02696-2, PMID: 34982230

[ref13] Delcher A. L. Bratke K. A. Powers E. C. Salzberg S. L. (2007). Identifying bacterial genes and endosymbiont DNA with glimmer. Bioinformatics 23, 673–679. doi: 10.1093/bioinformatics/btm009, PMID: 17237039 PMC2387122

[ref14] Dong H. H. Gao R. X. Dong Y. J. Yao Q. Zhu H. H. (2023). *Bacillus velezensis* RC116 inhibits the pathogens of bacterial wilt and fusarium wilt in tomato with multiple biocontrol traits. Int. J. Mol. Sci. 24:19. doi: 10.3390/ijms24108527, PMID: 37239871 PMC10217916

[ref15] Fan B. Wang C. Song X. F. Ding X. L. Wu L. M. Wu H. J.. (2019). *Bacillus velezensis* FZB42 in 2018: the gram-positive model strain for plant growth promotion and biocontrol (vol 9, 2491, 2018). Front. Microbiol. 10:1. doi: 10.3389/fmicb.2019.01279, PMID: 30386322 PMC6198173

[ref16] Federle M. J. (2009). Autoinducer-2-based chemical communication in bacteria: complexities of interspecies signaling. Contrib. Microbiol. 16, 18–32. doi: 10.1159/000219371, PMID: 19494577 PMC3042238

[ref17] Ferreira J. H. S. Matthee F. N. Thomas A. C. (1991). Biological-control of eutypa-lata on grapevine by an antagonistic strain of *Bacillus subtilis*. Phytopathology 81, 283–287. doi: 10.1094/Phyto-81-283

[ref18] Finn R. D. Clements J. Eddy S. R. (2011). HMMER web server: interactive sequence similarity searching. Nucleic Acids Res. 39, W29–W37. doi: 10.1093/nar/gkr367, PMID: 21593126 PMC3125773

[ref19] Galperin M. Y. Kristensen D. M. Makarova K. S. Wolf Y. I. Koonin E. V. (2019). Microbial genome analysis: the COG approach. Brief. Bioinform. 20, 1063–1070. doi: 10.1093/bib/bbx117, PMID: 28968633 PMC6781585

[ref20] Gershenzon J. Dudareva N. (2007). The function of terpene natural products in the natural world. Nat. Chem. Biol. 3, 408–414. doi: 10.1038/nchembio.2007.5 17576428

[ref21] Glickmann E. Dessaux Y. (1995). A critical-examination of the specificity of the salkowski reagent for indolic compounds produced by phytopathogenic bacteria. Appl. Environ. Microbiol. 61, 793–796. doi: 10.1128/aem.61.2.793-796.1995, PMID: 16534942 PMC1388360

[ref22] Goodwin S. McPherson J. D. McCombie W. R. (2016). Coming of age: ten years of next-generation sequencing technologies. Nat. Rev. Genet. 17, 333–351. doi: 10.1038/nrg.2016.49, PMID: 27184599 PMC10373632

[ref23] Hanif A. Zhang F. Li P. P. Li C. C. Xu Y. J. Zubair M.. (2019). Fengycin produced by *Bacillus amyloliquefaciens* FZB42 inhibits fusarium graminearum growth and mycotoxins biosynthesis. Toxins 11:11. doi: 10.3390/toxins11050295, PMID: 31137632 PMC6563212

[ref24] Harun-Or-Rashid M. Hwang J. H. Chung Y. R. (2017). Complete genome sequence of *Bacillus velezensis* YC7010, an endophytic bacterium with plant growth promoting, antimicrobial and systemic resistance inducing activities in rice. Korean J. Microbiol. 53, 329–331. doi: 10.7845/kjm.2017.7074

[ref25] He H. Cai X. Chen Y. Shen Z. Guan X. Hu F. (2002). Biological control of banana anthracnose with endophytic *Bacillus subtilis* BS-2 and BS-1 isolated from capsicum. J. Fujian Agric. For. Univ. 31, 442–444.

[ref26] Holtsmark I. Mantzilas D. Eijsink V. G. H. Brurberg M. B. (2006). Purification, characterization, and gene sequence of Michiganin a, an actagardine-like lantibiotic produced by the tomato pathogen *Clavibacter michiganensis* subsp michiganensis. Appl. Environ. Microbiol. 72, 5814–5821. doi: 10.1128/aem.00639-06, PMID: 16957199 PMC1563628

[ref27] Hotta K. Kim C. Y. Fox D. T. Koppisch A. T. (2010). Siderophore-mediated iron acquisition in bacillus anthracis and related strains. Microbiology 156, 1918–1925. doi: 10.1099/mic.0.039404-0 20466767

[ref28] Jiang C. H. Yao X. F. Mi D. D. Li Z. J. Yang B. Y. Zheng Y.. (2019). Comparative transcriptome analysis reveals the biocontrol mechanism of *Bacillus velezensis* F21 against *fusarium* wilt on watermelon. Front. Microbiol. 10:17. doi: 10.3389/fmicb.2019.00652, PMID: 31001229 PMC6456681

[ref29] Kazanas N. (1968). Proteolytic activity of microorganisms isolated from freshwater fish. Appl. Microbiol. 16, 128–132. doi: 10.1128/aem.16.1.128-132.1968, PMID: 5636454 PMC547330

[ref30] Kong W. J. Yan Y. C. Li X. Y. Liu Z. Y. (2018). Draft genome sequence of *Bacillus velezensis* PEBA20, a strain with a plant growth-promoting effect and biocontrol potential. Genome Announc. 6:2. doi: 10.1128/genomeA.00286-18, PMID: 29798912 PMC5968734

[ref31] Krzywinski M. Schein J. Birol I. Connors J. Gascoyne R. Horsman D.. (2009). Circos: an information aesthetic for comparative genomics. Genome Res. 19, 1639–1645. doi: 10.1101/gr.092759.109, PMID: 19541911 PMC2752132

[ref32] Kumar S. Stecher G. Tamura K. (2016). MEGA7: molecular evolutionary genetics analysis version 7.0 for bbigger datasets. Mol. Biol. Evol. 33, 1870–1874. doi: 10.1093/molbev/msw054, PMID: 27004904 PMC8210823

[ref33] Lagesen K. Hallin P. Rodland E. A. Staerfeldt H. H. Rognes T. Ussery D. W. (2007). RNAmmer: consistent and rapid annotation of ribosomal RNA genes. Nucleic Acids Res. 35, 3100–3108. doi: 10.1093/nar/gkm160, PMID: 17452365 PMC1888812

[ref34] Lam V. Meyer T. Arias A. A. Ongena M. Oni F. E. Hofte M. (2021). Bacillus cyclic lipopeptides iturin and fengycin control rice blast caused by pyricularia oryzae in potting and acid sulfate soils by direct antagonism and induced systemic resistance. Microorganisms 9:25. doi: 10.3390/microorganisms9071441, PMID: 34361878 PMC8305041

[ref35] Li L. Wang R. J. Liang X. X. Gai Y. P. Jiao C. Wang M. Q. (2023). Characterization of a *Bacillus velezensis* with antibacterial activity and its inhibitory eeffect on gray mold germ. Agronomy 13:21. doi: 10.3390/agronomy13061553

[ref36] Li P. Wang D. C. Yan J. L. Zhou J. A. Deng Y. Y. Jiang Z. D.. (2016). Genomic analysis of phylotype I strain EP1 reveals substantial divergence from other strains in the *ralstonia solanacearum* species complex. Front. Microbiol. 7:14. doi: 10.3389/fmicb.2016.01719, PMID: 27833603 PMC5080846

[ref37] Li S. Yang D. Qiu M. Shao J. Guo R. Shen B.. (2014). Complete genome sequence of *Paenibacillus polymyxa* SQR-21, a plant growth-promoting rhizobacterium with antifungal activity and rhizosphere colonization ability. Genome Announc. 2:e00281-14. doi: 10.1128/genomeA.00281-14, PMID: 24723719 PMC3983308

[ref38] Li Q. Zhang P. Liao B. Peng P. Mei J. Xu J. (2019). Isolation, identification and characterization of a cd resistant bacterium. Acta Microbiol Sin. 59, 11–24. doi: 10.13343/j.cnki.wsxb.20170632-en

[ref39] Li Y. Zhou Z. Yin X. (2021). Potential and genome-wide analysis of *Bacillus velezensis Mr12* in preventing apple ring rot and other diseases. J. Fruit Sci. 38, 1459–1467. doi: 10.13925/j.cnki.gsxb.20210129

[ref40] Liu G. Q. Kong Y. Y. Fan Y. J. Geng C. Peng D. H. Sun M. (2017). Whole-genome sequencing of *Bacillus velezensis LS69*, a strain with a broad inhibitory spectrum against pathogenic bacteria. J. Biotechnol. 249, 20–24. doi: 10.1016/j.jbiotec.2017.03.018, PMID: 28323017

[ref41] Loeffler W. Tschen J. S. M. Vanittanakom N. Kugler M. Knorpp E. Hsieh T. F.. (1986). Antifungal effects of bacilysin and fengymycin from *Bacillus subtilis* F-29-3 a comparison with activities of other bacillus antibiotics. J. Phytopathol. 115, 204–213. doi: 10.1111/j.1439-0434.1986.tb00878.x

[ref42] Lombard V. Ramulu H. G. Drula E. Coutinho P. M. Henrissat B. (2014). The carbohydrate-active enzymes database (CAZy) in 2013. Nucleic Acids Res. 42, D490–D495. doi: 10.1093/nar/gkt1178, PMID: 24270786 PMC3965031

[ref43] Lowe T. M. Eddy S. R. (1997). tRNAscan-SE: a program for improved detection of transfer RNA genes in genomic sequence. Nucleic Acids Res. 25, 955–964. doi: 10.1093/nar/25.5.955, PMID: 9023104 PMC146525

[ref44] Luo W. J. Liu L. D. Qi G. F. Yang F. Shi X. J. Zhao X. Y. (2019). Embedding *Bacillus velezensis* NH-1 in microcapsules for biocontrol of cucumber fusarium wilt. Appl. Environ. Microbiol. 85:13. doi: 10.1128/aem.03128-18, PMID: 30824441 PMC6495769

[ref45] Martinez-Raudales I. De La Cruz-Rodriguez Y. Vega-Arreguin J. Alvarado-Gutierrez A. Fraire-Mayorga A. Alvarado-Rodriguez M.. (2017). Draft genome sequence of *Bacillus velezensis* 3A-25B, a strain with biocontrol activity against fungal and oomycete root plant phytopathogens, isolated from grassland soil. Genome Announc. 5:e01021-17. doi: 10.1128/genomeA.01021-17, PMID: 28963212 PMC5624758

[ref46] McAuliffe O. Ross R. P. Hill C. (2001). Lantibiotics: structure, biosynthesis and mode of action. FEMS Microbiol. Rev. 25, 285–308. doi: 10.1111/j.1574-6976.2001.tb00579.x 11348686

[ref47] Medeiros C. A. A. Bettiol W. (2021). Multifaceted intervention of bacillus spp. against salinity stress and *fusarium* wilt in tomato. J. Appl. Microbiol. 131, 2387–2401. doi: 10.1111/jam.15095, PMID: 33817910

[ref48] Muttilainen S. Idanpaanheikkila I. Wahlstrom E. Nurminen M. Makela P. H. Sarvas M. (1995). The neisseia-meningitidis outer-membrane protein P1 produced in *Bacillus subtilis* and reconstituted into phospholipid-vesicles elicits antibodies to native P1 epitopes. Microb. Pathog. 18, 423–436. doi: 10.1006/mpat.1995.0038, PMID: 8551945

[ref49] Naglot A. Goswami S. Rahman I. Shrimali D. D. Yadav K. K. Gupta V. K.. (2015). Antagonistic potential of native trichoderma viride strain against potent tea fungal pathogens in north East India. Plant Pathol. J. 31, 278–289. doi: 10.5423/ppj.Oa.01.2015.0004, PMID: 26361476 PMC4564153

[ref50] Nannan C. Vu H. Q. Gillis A. Caulier S. Nguyen T. T. T. Mahillon J. (2021). Bacilysin within the *Bacillus subtilis* group: gene prevalence versus antagonistic activity against gram-negative foodborne pathogens. J. Biotechnol. 327, 28–35. doi: 10.1016/j.jbiotec.2020.12.017, PMID: 33387595

[ref51] Ongena M. Giger A. Jacques P. Dommes J. Thonart P. (2002). Study of bacterial determinants involved in the induction of systemic resistance in bean by *Pseudomonas putida* BTP1. Eur. J. Plant Pathol. 108, 187–196. doi: 10.1023/a:1015141503755

[ref52] Ongena M. Jacques P. (2008). Bacillus lipopeptides: versatile weapons for plant disease biocontrol. Trends Microbiol. 16, 115–125. doi: 10.1016/j.tim.2007.12.009, PMID: 18289856

[ref53] Ortiz A. Sansinenea E. (2020). Macrolactin antibiotics: amazing natural products. Mini-Rev. Med. Chem. 20, 584–600. doi: 10.2174/1389557519666191205124050 31804166

[ref54] Palazzini J. M. Dunlap C. A. Bowman M. J. Chulze S. N. (2016). *Bacillus velezensis* RC 218 as a biocontrol agent to reduce *fusarium* head blight and deoxynivalenol accumulation: genome sequencing and secondary metabolite cluster profiles. Microbiol. Res. 192, 30–36. doi: 10.1016/j.micres.2016.06.002, PMID: 27664721

[ref55] Patel P. S. Huang S. Fisher S. Pirnik D. Aklonis C. Dean L.. (1995). Bacillaene, a novel inhibitor of prokaryotic protein-synthesis produced by *Bacillus subtilis*-production, taxonomy, isolation, physicochemical characterization and biological-activity. J. Antibiot. 48, 997–1003. doi: 10.7164/antibiotics.48.997, PMID: 7592068

[ref56] Perry D. Abraham E. P. (1979). Transport and metabolism of bacilysin and other peptides by suspensions of *Staphylococcus aureus*. J. Gen. Microbiol. 115, 213–221. doi: 10.1099/00221287-115-1-213, PMID: 528972

[ref57] Petersen T. N. Brunak S. von Heijne G. Nielsen H. (2011). SignalP 4.0: discriminating signal peptides from transmembrane regions. Nat. Methods 8, 785–786. doi: 10.1038/nmeth.1701, PMID: 21959131

[ref58] Pleban S. Chernin L. Chet I. (1997). Chitinolytic activity of an endophytic strain of *Bacillus cereus*. Lett. Appl. Microbiol. 25, 284–288. doi: 10.1046/j.1472-765X.1997.00224.x, PMID: 9351279

[ref59] Rabbee M. F. Ali M. S. Choi J. Hwang B. S. Jeong S. C. Baek K. H. (2019). *Bacillus velezensis*: a valuable member of bioactive molecules within plant microbiomes. Molecules 24:13. doi: 10.3390/molecules24061046, PMID: 30884857 PMC6470737

[ref61] Song S. Wu P. Xing B. Gong G. (2011). Inhibitory effects and control efficacy of *Paenibacillus polymyxa* WY110 on watermelon *fusarium* wilt. Acta Phytophylacica Sin. 38, 571–572. doi: 10.13802/j.cnki.zwbhxb.2011.06.014

[ref62] Srinivas C. Devi D. N. Murthy K. N. Mohan C. D. Lakshmeesha T. R. Singh B.. (2019). *Fusarium oxysporum* f. sp. *lycopersici* causal agent of vascular wilt disease of tomato: biology to diversity-a review. Saudi J. Biol. Sci. 26, 1315–1324. doi: 10.1016/j.sjbs.2019.06.002, PMID: 31762590 PMC6864208

[ref63] Stoll A. Salvatierra-Martinez R. Gonzalez M. Araya M. (2021). The role of surfactin production by *Bacillus velezensis* on colonization, biofilm formation on tomato root and leaf surfaces and subsequent protection (ISR) against botrytis cinerea. Microorganisms 9:14. doi: 10.3390/microorganisms9112251, PMID: 34835375 PMC8626045

[ref1001] Teather R. M. Wood P. J. (1982). Use of congo red polysaccharide interactions in enumeration and characterization of cellulolytic bacteria from the bovine rumen. AEM 43, 777–780. 10.1128/aem.43.4.777-780.1982 PMC241917 7081984

[ref64] Wen T. Ding Z. X. Thomashow L. S. Hale L. Yang S. D. Xie P. H.. (2023). Deciphering the mechanism of fungal pathogen-induced disease-suppressive soil. New Phytol. 238, 2634–2650. doi: 10.1111/nph.18886, PMID: 36932631

[ref65] Wu J. J. Chou H. P. Huang J. W. Deng W. L. (2021). Genomic and biochemical characterization of antifungal compounds produced by *Bacillus subtilis* PMB102 against Alternaria brassicicola. Microbiol. Res. 251:126815. doi: 10.1016/j.micres.2021.126815, PMID: 34284299

[ref66] Wu L. M. Wu H. J. Chen L. Yu X. F. Borriss R. Gao X. W. (2015). Difficidin and bacilysin from *Bacillus amyloliquefaciens* FZB42 have antibacterial activity against *Xanthomonas oryzae* rice pathogens. Sci. Rep. 5:9. doi: 10.1038/srep12975, PMID: 26268540 PMC4534799

[ref67] Wu Y. Zhao C. Y. Farmer J. Sun J. D. (2015). Effects of bio-organic fertilizer on pepper growth and *fusarium* wilt biocontrol. Sci. Hortic. 193, 114–120. doi: 10.1016/j.scienta.2015.06.039

[ref68] Xie X. Zhang J. Wang H. Lei C. (2021). Research progress of the synthetic and functional mechanisms of natural lipopeptide antibiotics from bacillus. Chin. J. Antibiot. 46, 362–370. doi: 10.13461/j.cnki.cja.006979

[ref69] Xiong Q. Liu D. Zhang H. H. Dong X. Y. Zhang G. S. Liu Y. P.. (2020). Quorum sensing signal autoinducer-2 promotes root colonization of *Bacillus velezensis* SQR9 by affecting biofilm formation and motility. Appl. Microbiol. Biotechnol. 104, 7177–7185. doi: 10.1007/s00253-020-10713-w, PMID: 32621125

[ref70] Xu W. Yang Q. Yang F. Xie X. Goodwin P. H. Deng X. X.. (2022). Evaluation and genome analysis of *Bacillus subtilis* YB-04 as a potential biocontrol agent against *fusarium* wilt and growth promotion agent of cucumber. Front. Microbiol. 13:12. doi: 10.3389/fmicb.2022.885430, PMID: 35756052 PMC9218633

[ref71] Xu W. Zhang L. Y. Goodwin P. H. Xia M. C. Zhang J. Wang Q.. (2020). Isolation, identification, and complete genome assembly of an endophytic *Bacillus velezensis* YB-130, potential biocontrol agent against *fusarium* graminearum. Front. Microbiol. 11:12. doi: 10.3389/fmicb.2020.598285, PMID: 33343540 PMC7744476

[ref72] Xu Y. Zhang Y. Wang M. Wang S. Wang G. (2013). Research progress on bacteria quorum sensing. J. Henan Agric. Sci. 42, 16–19. doi: 10.15933/j.cnki.1004-3268.2013.12.004

[ref73] Xu T. Zhu T. Li S. Qiao T. (2014). Fungus-inhibitory activity and gene cloning ofbeta-glucanase from *Bacillus velezensis* YB15. Chin. J. Biol. Control 30, 276–281. doi: 10.16409/j.cnki.2095-039x.2014.02.019

[ref74] Yoon S. H. Ha S. M. Lim J. Kwon S. Chun J. (2017). A large-scale evaluation of algorithms to calculate average nucleotide identity. Anton. Leeuw. Int. J. Gen. Mol. Microbiol. 110, 1281–1286. doi: 10.1007/s10482-017-0844-4, PMID: 28204908

[ref75] Yuan J. Raza W. Shen Q. R. Huang Q. W. (2012). Antifungal activity of *Bacillus amyloliquefaciens* NJN-6 volatile compounds against *fusarium oxysporum* f. sp *cubense*. Appl. Environ. Microbiol. 78, 5942–5944. doi: 10.1128/aem.01357-12, PMID: 22685147 PMC3406121

[ref76] Zhang D. Gao Y. Wang Y. Liu C. Shi C. (2020a). Advances in taxonomy, antagonistic function and application of *Bacillus velezensis*. Microbiol. China 47, 3634–3649. doi: 10.13344/j.microbiol.china.190947

[ref77] Zhang D. Yu S. Q. Yang Y. Q. Zhang J. L. Zhao D. M. Pan Y.. (2020b). Antifungal effects of volatiles produced by *Bacillus subtilis* against alternaria solaniin potato. Front. Microbiol. 11:12. doi: 10.3389/fmicb.2020.01196, PMID: 32625175 PMC7311636

[ref78] Zhang Q. Zabihullah S. Tang C. (2020). Biocontrol effect of *Bacillus velezensis* strain SZAD1 on verticillium dahliae. Cotton Sci. 32, 329–338. doi: 10.11963/1002-7807.zqtcm.20200609

[ref79] Zhao J. Mei Z. Zhang X. Xue C. Zhang C. Z. Ma T. F.. (2017). Suppression of *fusarium* wilt of cucumber by ammonia gas fumigation via reduction of *fusarium* population in the field. Sci. Rep. 7, 1–8. doi: 10.1038/srep43103 28230182 PMC5322401

[ref80] Zhao X. H. Xu Y. L. Viel J. H. Kuipers O. P. (2021). Semisynthetic macrocyclic lipo-lanthipeptides display antimicrobial activity against bacterial pathogens. ACS Synth. Biol. 10, 1980–1991. doi: 10.1021/acssynbio.1c00161, PMID: 34347446 PMC8383303

[ref81] Zheng J. F. Ge Q. W. Yan Y. C. Zhang X. P. Huang L. Yin Y. B. (2023). dbCAN3: automated carbohydrate-active enzyme and substrate annotation. Nucleic Acids Res. 51, W115–W121. doi: 10.1093/nar/gkad328, PMID: 37125649 PMC10320055

[ref82] Zhou Z. C. Tang X. Y. Peng L. J. Ding H. X. (2023). Complete genome sequence of *Bacillus velezensis* GUAL210, a potential biocontrol agent isolated from pepper rhizosphere. Plant Dis. 107, 915–918. doi: 10.1094/pdis-07-22-1585-a, PMID: 36265149

[ref83] Zhou J. Y. Wang M. Sun Y. M. Gu Z. C. Wang R. R. Saydin A.. (2017). Nitrate increased cucumber tolerance to *fusarium* wilt by regulating fungal toxin production and distribution. Toxins 9:20. doi: 10.3390/toxins9030100, PMID: 28287458 PMC5371855

[ref84] Zhu L. Y. Liu N. Wang H. Q. Zhang Z. D. Jiang L. Huang H. (2019). Draft genome sequence of broad-spectrum antifungal-producing *Bacillus velezensis* C4341 isolated from a saline-alkali soil sample in China. J. Glob. Antimicrob. Resist. 16, 291–293. doi: 10.1016/j.jgar.2018.12.019, PMID: 30802554

[ref85] Zong Y. Zhao Y. Liu Y. Yang Q. (2018). Study on the inhibitory effect of *Bacillus velezensis* on *fusarium* graminearum. J. Nuclear Agric. Sci. 32, 310–317. doi: 10.11869/j.issn.100-8551.2018.02.0310

