# Supplementary figures and images for: Genome sequencing and analysis of Bacillus velezensis VJH504 reveal biocontrol mechanism against cucumber Fusarium wilt
Source: Front Microbiol. 2023 Oct 12;14:1279695. doi: 10.3389/fmicb.2023.1279695 (PMC10602789; doi:10.3389/fmicb.2023.1279695)

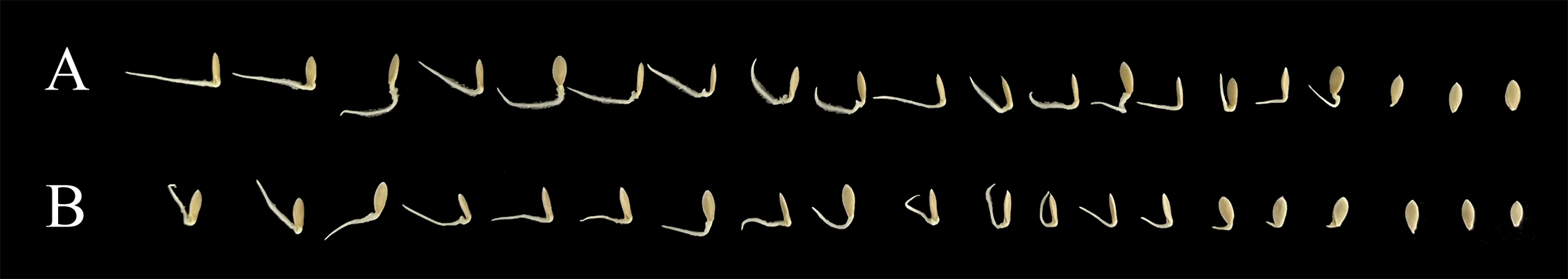

Supplement: Supplementary file 1 [file Image_1.TIF]
